# A Surface Engineering Strategy to Suppress Cathodic Precipitation in Seawater Electrolysis

**DOI:** 10.1002/smsc.70368

**Published:** 2026-08-12

**Authors:** Vasundhara Nettem, Muhammad Waqas Khan, Suraj Loomba, Sharafadeen Gbadamasi, Tushar Verma, Babar Shabbir, Ravichandar Babarao, Nasir Mahmood

**Affiliations:** ^1^ School of Science RMIT University Melbourne Victoria Australia; ^2^ Academy of Scientific and Innovative Research (AcSIR) Ghaziabad India

**Keywords:** ammonia modification, catalyst durability, cathodic precipitation, hydrogen evolution reaction, interfacial layer, seawater splitting

## Abstract

Electrochemical seawater splitting powered by renewable energy offers a pathway to green hydrogen, but cathodic precipitation of Mg/Ca hydroxides severely impairs kinetics and deactivates catalysts. Here, we develop an ammonia‐modified Cu_3_P/MoP (A‐Cu_3_P/MoP) heterostructure that delivers efficient and precipitation‐resistant hydrogen evolution in natural and alkaline seawater. Density functional theory calculations reveal that surface NH_4_^+^ species dramatically reduce the overall free energy of reaction intermediates and lower the potential‐determining step to 1.24 eV at the *H—OH transition, thereby facilitating the Volmer step. Benefiting from this optimized interfacial chemistry, A‐Cu_3_P/MoP achieves 0.50 A cm^−2^ at 574 mV in natural seawater and 1.0 A cm^−2^ at 416 mV in alkaline seawater, outperforming Pt/C. The catalyst maintains stable operation for over 500 h at 100 mA cm^−2^, whereas Pt/C rapidly degrades within 50 h due to severe Mg/Ca hydroxide deposition. In a 25 cm^2^ zero‐gap electrolyzer paired with a lab‐designed anode, A‐Cu_3_P/MoP sustains ~420 mA cm^−2^ at 1.23 V for more than 50 h in seawater with negligible performance loss. Mechanistic studies indicate that interfacial NH_4_^+^ forms a positively charged layer that electrostatically repels Mg^2+^/Ca^2+^, suppressing hydroxide precipitation and enabling durable seawater electrolysis.

## Graphical abstract

Ammonia‐modified Cu_3_P/MoP heterostructures confine NH^4+^ at the cathode, creating a positively charged layer that repels Mg^2+^/Ca^2+^ and suppresses hydroxide precipitation. DFT reveals enhanced charge redistribution at Mo sites, lowering the water‐dissociation barrier. The catalyst achieves 0.50 A cm^−2^ at 574  mV in natural seawater and stable hydrogen evolution over 500 h.

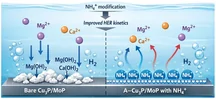

## 1 Introduction

Hydrogen production via electrolysis of seawater offers a practical alternative to reduce reliance on freshwater [1, 2]. As a key strategy for large‐scale decarbonization, seawater electrolysis can supply sustainable hydrogen to support the global energy transition away from fossil‐fuel dependence. In centralized systems, the direct cost impact of desalination on the levelised cost of hydrogen may be limited. Yet the requirement for treated freshwater, storage and distribution infrastructure adds to capital expenditure and constrains siting flexibility. Decentralized seawater electrolysis, which exploits locally available seawater, can mitigate these constraints by reducing freshwater demand and the need for extensive water‐supply infrastructure. Seawater is abundant and readily accessible, making it an attractive electrolyte for distributed hydrogen generation [3, 4]. Nevertheless, the direct use of seawater poses complex operational challenges due to its high ionic content and impurities that compromise electrode stability and catalytic activity [5, 6].

A major challenge encountered during the hydrogen evolution reaction (HER) in seawater electrolysis is cathodic precipitation, particularly under high current densities [7, 8]. As water molecules approach the cathode and undergo reduction, protons are adsorbed on the electrode surface, while hydroxide ions (OH^−^) are generated and accumulate in the vicinity of the cathode [9, 10]. This local enrichment of hydroxides significantly alters the microenvironment. The rate of OH^−^ accumulation is faster than the rate at which it diffuses away, and the electrode surface becomes highly alkaline, promoting unwanted precipitation [4, 11, 12]. Hydroxide ions possess a strong affinity for divalent metal cations, notably Mg^2+^ and Ca^2+^ ions, which are present at substantial concentrations in natural seawater [13, 14]. When these cations interact with excess OH^−^ near the cathode, they readily precipitate as insoluble hydroxides, such as Mg (OH)_2_ and Ca (OH)_2_, forming dense, white deposits on the electrode surface. This precipitation passivates the active catalytic sites, severely impeding electron transfer and drastically compromising HER efficiency and long‐term stability [15, 16]. While alkaline seawater can reduce the concentrations of these cations, even trace amounts remaining are sufficient to trigger precipitation under operating conditions, at high current densities [17, 18]. During vigorous electrolysis, the continuous generation and accumulation of OH^−^ are unavoidable, sustaining conditions conducive to ongoing precipitation [19, 20]. This persistent precipitation on the cathode not only mandates frequent maintenance and replacement of electrodes but also presents a fundamental barrier to scaling seawater electrolysis for practical hydrogen production [21, 22]. Addressing cathodic precipitation, therefore, is essential for unlocking both the efficiency and durability of sustainable seawater‐based hydrogen generation.

Traditional state‐of‐the‐art platinum‐based catalysts falter under such harsh conditions, being quickly deactivated by cathodic precipitation [23, 25]. As the search for resilient, efficient, and low‐cost alternatives intensifies, transition metal phosphides (TMPs) have garnered attention as promising HER catalysts [26, 28]. Their favorable electronic structures, strong P‐mediated conductivity, and robust alkaline stability render TMPs like Cu_3_P and MoP viable nonprecious‐metal alternatives to platinum [29, 30]. Nonetheless, single‐component phosphides often suffer from limited activity or durability when exposed to real seawater conditions [31, 32]. Forming Cu_3_P/MoP heterostructure enables synergistic coupling where Cu sites could enhance charge transport and Mo sites optimize water dissociation and hydrogen binding to boost catalytic activity. However, mitigating hydroxide precipitation remains a significant challenge to be addressed [33].

In this work, we report the rational design and investigation of an ammonia‐modified Cu_3_P/MoP (A‐Cu_3_P/MoP) heterostructure for natural and alkaline seawater electrolysis. By confining NH_4_^+^ species at the catalyst interface, our approach creates a positively charged interfacial layer that repels metal cations, effectively suppressing the precipitation of Mg(OH)_2_ and Ca(OH)_2_. While NH_4_^+^ has previously been implicated in mitigating cathodic precipitation via in situ ammonium generation at a Mo_2_N catalyst surface, where dynamically formed NH_4_^+^ suppresses local pH increase [11]. Our strategy instead uses ex situ, preinstalled NH_4_^+^ that electrostatically repels Mg^2+^/Ca^2+^ cations directly, rather than suppressing the pH rise that drives precipitation. Density functional theory (DFT) calculations further reveal that these surface‐bound ammonia species enhance charge redistribution at Mo‐centered active sites. This lowers the water dissociation barrier and thermodynamically stabilizes hydrogen intermediates, jointly promoting HER kinetics and durability (>500 h). As a result, A‐Cu_3_P/MoP catalyst achieves outstanding and long‐lasting HER performance, delivering current densities of 0.50 A cm^−2^ at an overpotential of 574 mV in natural seawater and an even higher 1.0 A cm^−2^ at 416 mV in alkaline seawater for HER with a Tafel slope of 33.5 mV dec^−1^ in challenging seawater environments, demonstrating a promising pathway for surmounting the long‐standing limitations of direct seawater electrolysis.

## 2 Experimental Section

### 2.1 Synthesis of Ammonia‐Modified Cu_3_P/MoP Sheets

In a typical synthesis process, 100 mg of copper (II) nitrate trihydrate is dispersed in 10 mL of ethanol, and 100 mg of ammonium molybdate tetrahydrate is dissolved in 10 mL of distilled water to obtain a homogeneous solution. For the precursor over template system, the obtained precursor solutions were added dropwise to 310 g of NaCl salt (template) and mixed for 20 min at 80 °C on a hotplate. This precursor over template was annealed in a tube furnace under N_2_ at 700 °C for 1 h to obtain the 2D CuMo oxide after filtration. The obtained oxide sample was phosphorised at 350 °C with a heating rate of 3 °C min^−1^ under N_2_ for 3 h. Sodium hypophosphite was used as the phosphorus source, maintaining a ratio of 1:30 between the sample and the P source. To introduce ammonia into the Cu_3_P/MoP heterostructure, the phosphorised sample was immersed in a commercial ammonium hydroxide solution (28–30 wt% NH_3_, approximately 14.5–15 M at room temperature), which was used without further dilution for the surface modification treatment allowed to soak for 12 h at room temperature. Following the treatment, the sample was carefully dried and subsequently annealed at 250 °C for 2 h under an inert (N_2_) atmosphere.

### 2.2 Electrochemical Testing

Electrochemical measurements were carried out at room temperature using an electrochemical workstation (Corrtest) in a conventional three‐electrode configuration, with 1 M alkaline seawater as the electrolyte. Natural seawater was collected from Altona Beach, Melbourne, Australia, filtered to remove suspended impurities, and then mixed with the required amount of KOH to obtain a 1 M alkaline seawater solution. The electrolyte was allowed to stand overnight, after which the clear supernatant was collected for electrochemical testing. A graphite rod and an Hg/HgO electrode were used as the counter and reference electrodes, respectively. The working electrodes were fabricated by drop‐casting the catalyst ink onto pretreated nickel foam substrates. Before use, the nickel foam was sequentially cleaned with 1 M HCl, deionized water, and ethanol. To prepare the catalyst ink, a carbon black (CB) dispersion was first obtained by sonicating 20 mg of carbon powder in 20 mL of a mixed solvent consisting of isopropyl alcohol and water (1:4, v/v) for 1 h. Subsequently, 4 mg of the catalyst, 1 mL of the CB dispersion, and 8 µL of polytetrafluoroethylene (PTFE) binder were combined and homogenized by ultrasonication for 30 min. An aliquot of 200 µL of the resulting ink was drop‐cast onto a 0.25 cm^2^ area of the cleaned nickel foam and dried in a vacuum oven at 60 °C for 12–24 h. Linear sweep voltammetry (LSV) measurements were conducted without iR compensation. All measured potentials were converted to the reversible hydrogen electrode (RHE) scale using the following relationship *E* (*V* vs RHE) = *E*_(Hg/HgO)_ + (0.059 * pH) + 0.098.

## 3 Results and Discussion

**FIGURE 1:**
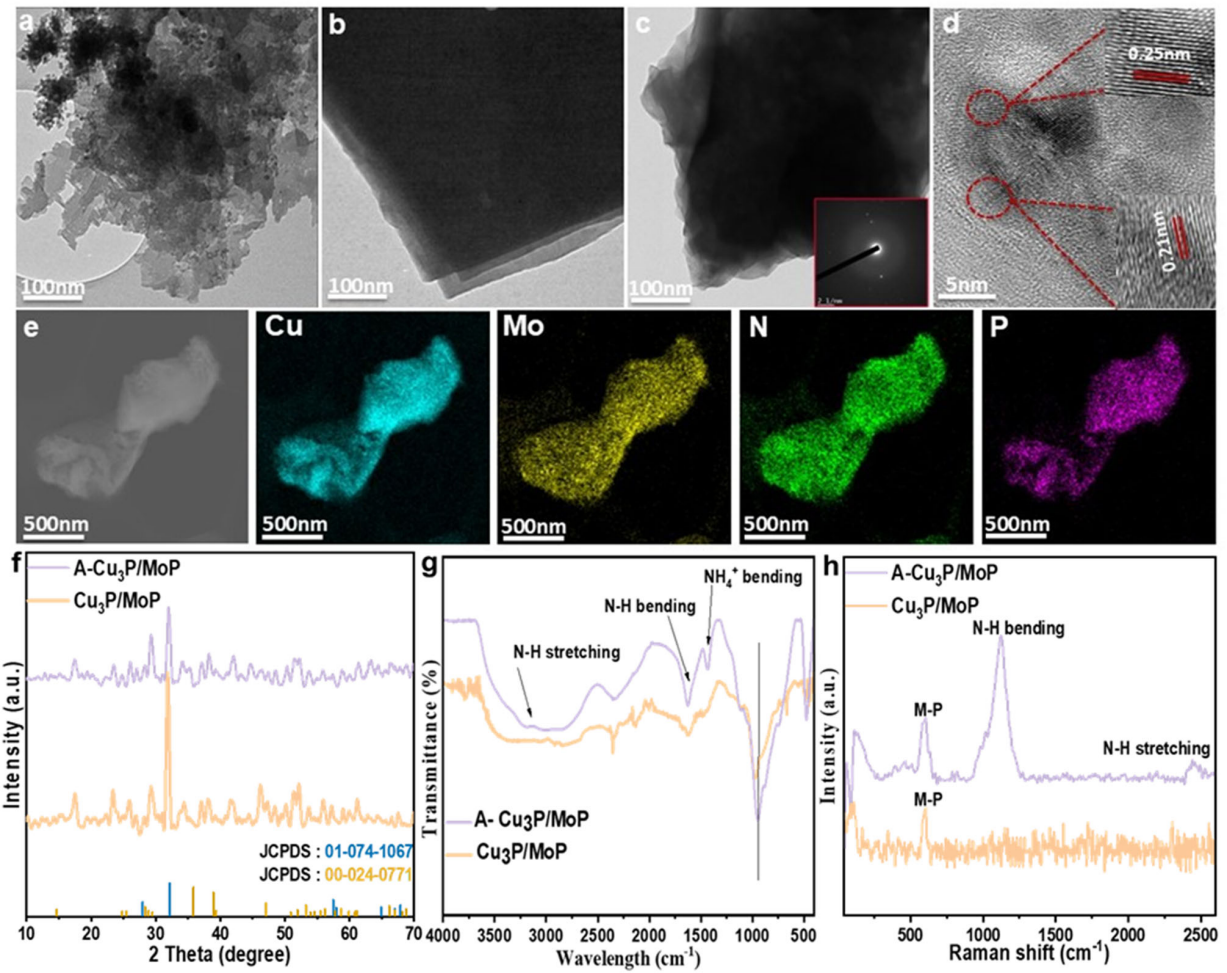
(a) TEM image of CuMoO_4_, (b) TEM image of Cu_3_P/MoP, (c) TEM image of A‐Cu_3_P/MoP (subset: SAED), (d) HR‐TEM of A‐Cu_3_P/MoP, Lattice fringes of Cu_3_P, MoP, (e) EDS elemental mapping of Cu, Mo, N, P of A‐Cu_3_P/MoP, (f) XRD of A‐Cu_3_P/MoP and Cu_3_P/MoP, (g) FT‐IR of A‐Cu_3_P/MoP and Cu_3_P/MoP, and (h) Raman spectra of A‐Cu_3_P/MoP and Cu_3_P/MoP.

To reduce cathodic precipitation in seawater, an ammonia‐modified Cu_3_P/MoP (A‐Cu_3_P/MoP) catalyst is developed through a sequential oxideto phosphides followed by ammonium ion surface modification, as depicted in Figure S1. The transformation pathway of the heterostructure is clearly illustrated by transmission electron microscopy (TEM) images across the synthesis stages. The initial CuMoO_4_ oxide precursor (Figure 1a) reveals a distinct sheet‐like morphology, which is largely improved after conversion to Cu_3_P/MoP (Figure 1b). Additional TEM images of CuO and Cu_3_P prior to heterostructure formation, presented in the Figures S2–S4, further confirm the continuity of nanosheet architecture across all transformation steps. Following ammonia modification, the sheet structure remains evident in the A‐Cu_3_P/MoP (Figure 1c), indicating that the entire synthesis pathway preserves the 2D sheet morphology. The corresponding Selected area elctron diffraction (SAED) displays weak diffraction spots, indicating only limited crystalline regions (the inset of Figure 1c). The high‐resolution TEM (HRTEM) image reveals short‐range lattice fringes with interplanar spacings of approximately 0.25 and 0.21 nm (Figure 1d), attributable to the (112) plane of Cu_3_P and the (101) plane of MoP, respectively.

Taken together, SAED and HRTEM indicate a structurally integrated heterostructure in which nanocrystallite domains of Cu_3_P and MoP are clearly found. Furthermore, elemental mapping by energy‐dispersive X‐ray spectroscopy (Figure 1e) demonstrates that Cu, Mo, P, and N are homogeneously distributed across the 2D network. This spatial uniformity confirms successful integration of the phosphide phases and the effective incorporation of ammonia‐derived nitrogen species on the catalyst surface.

Further, the powder X‐ray diffraction peaks confirm the presence of hexagonal Cu_3_P (JCPDS No. 01‐074‐1067) and MoP (JCPDS No. 00‐024‐0771), verifying the formation of the targeted heterostructure Cu_3_P/MoP (Figure 1f). In contrast to the sharp and well‐defined peaks seen for the precursor CuO and the single‐phase Cu_3_P (Figures S5 and S6), the MoP phase in Cu_3_P/MoP shows no prominent diffraction peaks, indicating that its crystallinity is relatively low [33]. This behavior reflects the presence of MoP in a highly dispersed nanocrystalline form confined at the heterointerface, where Mo remains structurally dominant despite reduced bulk crystallinity [33]. In A‐Cu_3_P/MoP, the diffraction peaks of the catalyst become even broader and less intense, suggesting a further decrease in crystallinity coherence. This reduction is consistent with the amorphous features observed by HRTEM and is likely induced by the introduction of surface‐bound ammonium species. FT‐IR spectroscopy further confirms the successful surface modification as A‐Cu_3_P/MoP displayed characteristic N—H stretching bands appearing at 3200–3400 cm^−1^, NH_4_^+^ bending around 1400 cm^−1^, and pronounced N—H bending vibrations near 1600 cm^−1^ (Figure 1g) [34, 36]. Absorption features in the 900–1000 cm^−1^ region are assigned to the Cu_3_P/MoP framework, confirming the retention of essential structural components after modification [35]. Raman spectroscopy further validates the incorporation of ammonia‐derived surface species in A‐Cu_3_P/MoP. A broad peak observed around 1160 cm^−1^ can be attributed to N—H bending vibrations (Figure 1h) [37]. Additionally, a prominent peak around 2455 cm^−1^ was assigned to an N—H stretching, which is consistent with the incorporation of ammonium ions on the surface [38].

**FIGURE 2:**
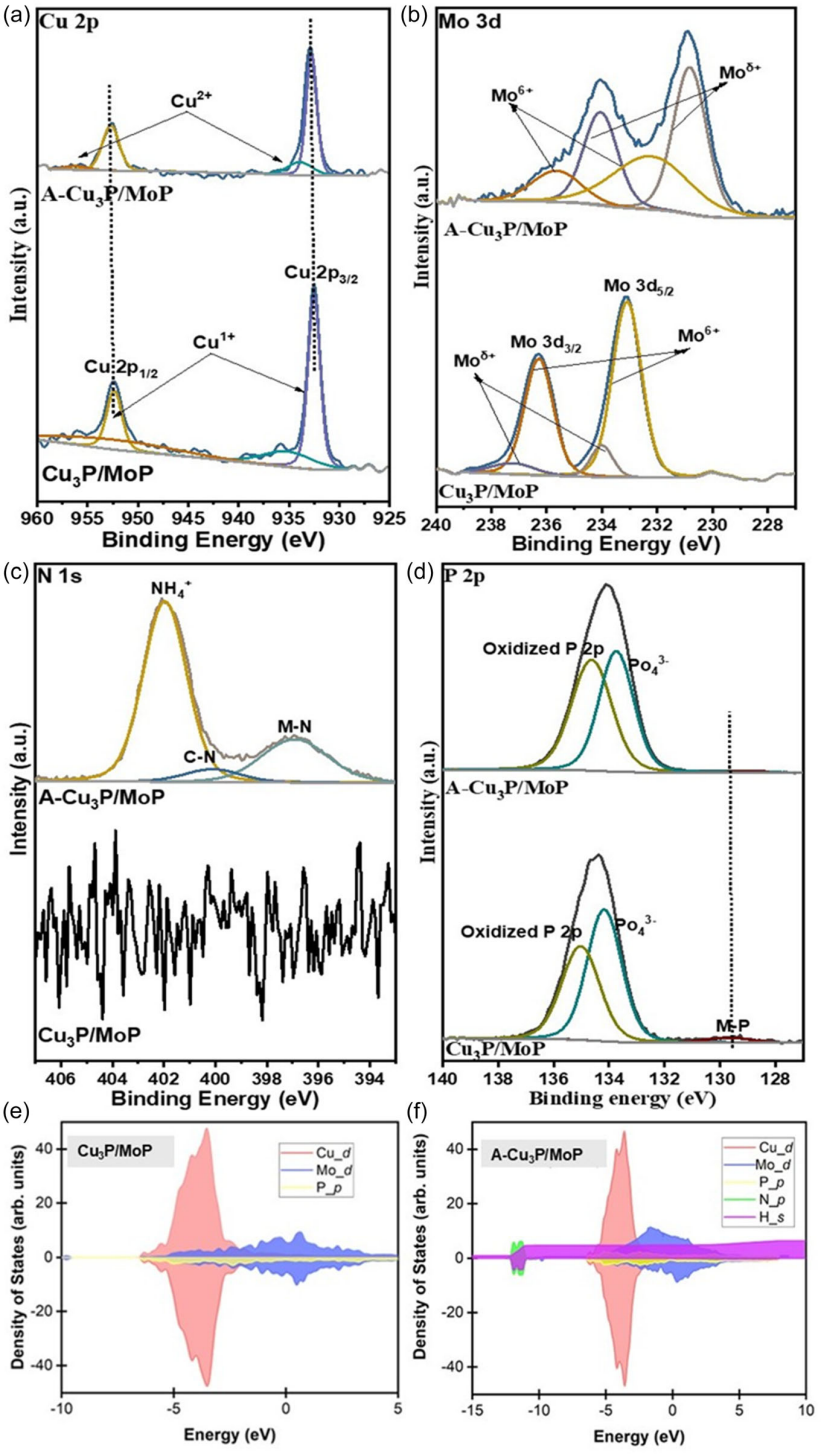
XPS analysis of A‐Cu_3_P/MoP (a) Cu 2p, (b) Mo 3d, (c) N 1s, (d) P 2p, (e) DOS plots of Cu_3_P‐MoP, and (f) DOS plots of A‐Cu_3_P‐MoP heterosurface.

X‐ray photoelectron spectroscopy (XPS) analysis provides critical insights into the electronic interactions and bonding environments in the Cu_3_P/MoP catalyst following ammonia modification (Figures 2 and S7–S9). The Cu 2p spectra display peaks at 932.89 and 952.31 eV, which are assigned to Cu 2p_3/2_ and Cu 2p_1/2_, respectively, of Cu^+^ of Cu_3_P (Figure 2a) [39]. After ammonia modification, the Cu 2p spectra of A‐Cu_3_P/MoP areessentially unchanged, indicating the Cu—P environment is preserved and that Cu sites are only weakly perturbed by NH_4_^+^ adsorption. In contrast, the Mo 3d spectra (Figure 2b) reveal substantial changes in the electronic environment. In Cu_3_P/MoP, Mo 3d peaks appear at 233.10 and 236.29 eV (Mo 3d_5/2_ and Mo 3d_3/2_), which are characteristic of oxidized Mo species, along with additional peaks at 234.01 and 237.23 eV. These high binding energies suggest the presence of surface Mo—O bonds originating from partial oxidation of MoP [40]. After ammonia functionalization, however, the dominant Mo 3d peaks shift to 230.82 and 234.01 eV, accompanied by the 234.29 and 237.23 eV components [41]. The pronounced negative shift of approximately 2.28 eV for the main Mo 3d_5/2_ and Mo 3d_3/2_ peaks, considering that ammonia acts as a reducing agent, the observed shift is attributed to the reduction of surface Mo—O species and the formation of more electron rich Mo—P environments at Mo centers, reflects surface reconstruction. Precisely, NH_4_^+^ selectively binds to and restructures the more oxophilic Mo sites, while the Cu^+^ sites remain largely unaffected. This significant shift is indicative of electron enrichment at Mo centers and is beneficial for HER because it lowers the energy barrier for water dissociation and stabilizes key hydrogen intermediates at Mo active sites, as further supported by the DFT results discussed below.

The N 1s spectra (Figure 2c) provide further evidence for surface modification. In Cu_3_P/MoP, no N 1s signal is detected, confirming the absence of nitrogen‐containing species. After ammonia modification (A‐Cu_3_P/MoP), three distinct peaks emerge at 401.96, 400.11, and 396.92 eV. The high binding energy peak at 401.96 eV is attributed to NH_4_^+^ ions, confirming successful ammonium adsorption onto the catalyst surface [42, 43]. Additional features at other binding energies (400.11 and 396.92 eV) in the N 1s region suggest multiple nitrogen coordination states on the surface, including possible interactions with copper, further supporting the formation of metal—N bonds [42]. This evidence points to strong adsorption and interaction of NH_4_^+^ at the surface, which facilitates charge redistribution and enhances electron density specifically at Mo active sites. The P 2p spectra (Figure 2d) reinforce the stability of the phosphide framework, with a distinct phosphide peak observed at 129.3 eV, characteristic of P in metal—phosphide bonds [44]. No significant differences are detected after ammonia modification, indicating that the phosphidic environment remains stable and unaffected. The heatmap of binding energy shifts (Figure S10) summarizes these findings: Cu and P remain unchanged, Mo exhibits a strong negative shift (−2.28 eV), and N 1s appears newly, confirming NH_4_^+^ adsorption. Hence, it is established that electron flow occurs from NH_4_^+^ → Mo (primary direction), with Cu and P as stable supporting sites.

To rationalize these observations, the projected density of states (PDOS) and charge‐density‐difference analyses were performed for Cu_3_P/MoP and A‐Cu_3_P/MoP (Figure 2e,f). The PDOS of pristine Cu_3_P/MoP shows that states near the Fermi level are mainly derived from Cu *d* and Mo *d* orbitals, with only a modest contribution from P *p* states, reflecting relatively localized metal—P bonding and limited nonmetal participation in charge transport. When NH_4_^+^ is introduced, N *p* and H *s* orbitals strongly hybridize with Cu *d*, Mo *d*, and P *p* states near the Fermi level, producing a broader and more continuous electronic distribution that facilitates rapid electron transfer and adsorbate activation. Consistently, the charge‐density‐difference isosurfaces reveal charge donation from NH_4_^+^ to the Cu_3_P/MoP surface and electron accumulation predominantly around Mo—P units, corroborating the XPS‐derived picture of NH_4_^+^ to Mo electron flow and interfacial electronic enrichment that underpins the enhanced HER kinetics.

**FIGURE 3:**
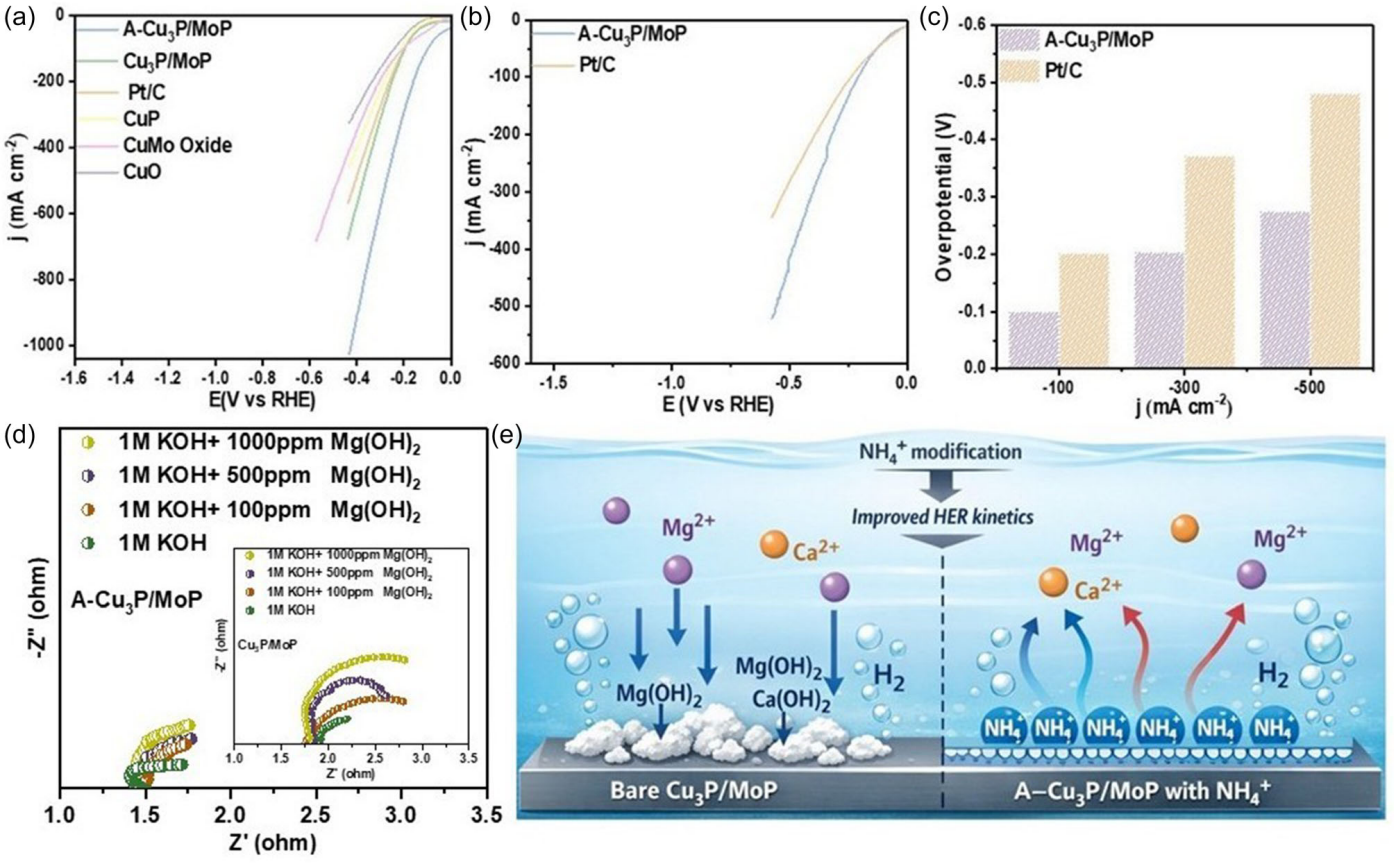
(a) LSV measurements of A‐Cu_3_P/MoP, Cu_3_P/MoP, Pt/C, CuP, CuMo oxide, and CuO, (b) LSV of A‐Cu_3_P/MoP, Pt/C in natural seawater, and (c) comparison of the overpotentials achieved by A‐Cu_3_P/MoP, Cu_3_P/MoP, in 1 M alkaline seawater electrolyte at different current densities. (d) Nyquist plots of A‐Cu_3_P/MoP and Cu_3_P/MoP(inset) electrodes measured in 1 M KOH electrolyte supplemented with increasing concentrations of Mg(OH)_2_ (100, 500, and 1000 ppm). (e) Schematic of Cu_3_P/MoP/A‐Cu_3_P/MoP with precipitation mechanism (image illustration generated with AI‐assisted image generation, manually reviewed and validated by all authors for scientific accuracy prior to inclusion).

To elucidate the influence of ammonium ions on the HER, electrochemical tests were conducted in 1 M alkaline seawater. LSV revealed that, among CuO (*η*_100_ = 240 mV), CuP (*η*_100_ = 210 mV), CuMo oxide (*η*_100_ = 202 mV), Cu_3_P/MoP (*η*_100_ = 201 mV), and Pt/C (*η*_100_ = 200 mV), the ammonia‐modified Cu_3_P/MoP (*η*_100_ = 97 mV) exhibited the best activity, exceeding even commercial Pt/C (Figure 3a). To probe the effect of ammonia concentration, LSV measurements were performed on Cu_3_P/MoP treated with 0.5, 1, 2, 5 M, and saturated ammonia solutions (Figure S11). The HER performance exhibited a concentration‐dependent enhancement, with the saturated ammonia treatment achieving the highest activity. The improvement can be attributed to surface reconstruction and electronic modulation induced by NH_4_^+^ ions. Further, in natural seawater, the A‐Cu_3_P/MoP reached a current density of 0.50 A cm^−2^ at 574 mV overpotential (Figure 3b), clearly highlighting the dominance of ammonia‐based modification in mitigating seawater harshness. In alkaline seawater, A‐Cu_3_P/MoP achieved current densities of 100, 500, and 1000 mA cm^−2^ at even low overpotentials of 97, 274, and 426 mV, respectively (Figure 3c). Ammonium ions enhance performance by forming nitrogen‐containing surface species that modulate local coordination and active‐site density, thereby promoting faster proton adsorption and charge transfer.

The Tafel slope (Figure S12) shows that A‐Cu_3_P/MoP has the smallest slope, 33.5 mV dec^−1^, outperforming Cu_3_P/MoP (43.7 mV dec^−1^) and Pt/C (73.5 mV dec^−1^), indicating more favorable HER kinetics on the ammonia‐modified surface. A Tafel slope close to 30 mV dec^−1^ suggests that the Volmer step is fast and that the Volmer–Heyrovsky sequence is likely rate‐determining under alkaline conditions [29]. Electrochemical impedance spectroscopy (Figure S13) further shows a markedly reduced charge‐transfer resistance for A‐Cu_3_P/MoP relative to Cu_3_P/MoP and Pt/C, confirming improved interfacial conductivity and faster electron transport. Double‐layer capacitance (*C*_dl_) analysis by cyclic voltammetry (Figure S14) revealed an Elctrochemical surface area (ECSA) of 55 mF cm^−2^ for A‐Cu_3_P/MoP, over twice that of Pt/C (24 mF cm^−2^), highlighting a higher density of electrochemically accessible active sites. Collectively, the interplay of improved surface chemistry, electronic structure, charge transfer, and ECSA yields exceptional HER performance in alkaline seawater. The beneficial role of ammonium ions can be rationalized by several factors. First, ammonia modification likely promotes the formation of nitrogen‐containing surface species (adsorbed NH_3_), which modulate the local coordination environment, increase the density of catalytically active sites, and facilitate proton adsorption. Second, NH_4_^+^ ions subtly modify the electronic structure at the Cu_3_P/MoP interface, thereby enhancing charge transfer kinetics and lowering the energy barrier for hydrogen evolution. Third, the positively charged NH_4_^+^ layer provides an electrostatic shield that repels Mg^2+^/Ca^2+^, mitigates cathodic precipitation, and helps maintain these optimized active sites during prolonged operation in seawater.

To directly evaluate the resistance of A‐Cu_3_P/MoP toward Mg^2+^ interference, Electrochemical impendance spectroscopy (EIS) was carried out in 1 M KOH with increasing concentrations of 100, 500, and 1000 ppm of Mg(OH)_2_ (Figure 3d). For Cu_3_P/MoP (inset), the Nyquist plots show a systematic increase in semicircle diameter with increasing Mg(OH)_2_ concentration, indicating a concentration‐dependent increase in charge‐transfer resistance (*R*_ct_). In stark contrast, A‐Cu_3_P/MoP exhibits a largely invariant semicircle diameter across the entire Mg^2+^ concentration range tested, with Rct remaining essentially unchanged from the pure KOH baseline. This concentration‐independent response directly confirms that the surface‐confined NH_4_^+^ layer effectively shields the active sites from Mg^2+^ interference through electrostatic repulsion. The absence of any low‐frequency mass‐transport arc in A‐Cu_3_P/MoP, even at 1000 ppm Mg(OH)_2_, further confirms that precipitation‐driven diffusional limitations, which begin to emerge in Cu_3_P/MoP at elevated concentrations, are entirely prevented by the ammonium‐modified interface. To visualize the role of ammonium ions in suppressing cathodic precipitation, schematic models are presented in Figure 3e. It shows that the bare Cu_3_P/MoP surface is directly exposed to Mg^2+^ and Ca^2+^, allowing these cations to approach the cathode and form insulating Mg (OH)_2_/Ca(OH)_2_ precipitates that block active sites and deteriorate HER performance. In contrast, the right side of the figure illustrates the A‐Cu_3_P/MoP surface, where surface‐confined NH_4_^+^ species create a positively charged interfacial “shield” that electrostatically repels Mg^2+^/Ca^2+^ and inhibits metal hydroxide deposition. This illustration is consistent with the enhanced activity, lower charge‐transfer resistance, and excellent durability observed electrochemically for the ammonia‐modified catalyst in seawater.

**FIGURE 4:**
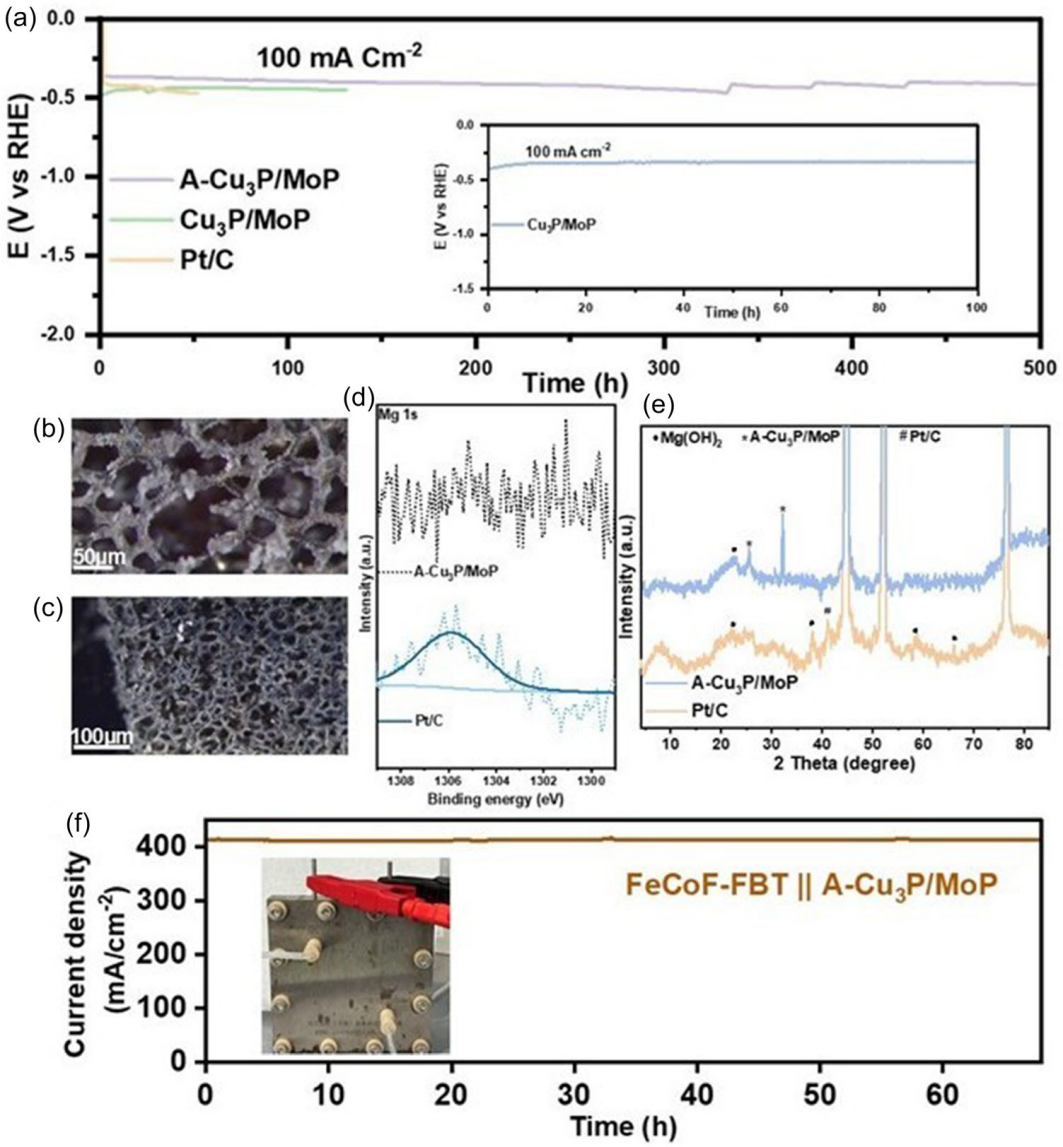
(a) Chronopotentiometry of A‐Cu_3_P/MoP, Cu_3_P/MoP, Pt/C in alkaline seawater at 100 mA cm^−2^ current density, Inset chronopotentiometry of A‐Cu_3_P/MoP in natural seawater at 100 mA cm^−2^ current density, (b) optical images of Pt/C, (c) optical image of A‐Cu_3_P/MoP, (d) ex situ XPS of Mg 1s, (e) XRD analysis A‐Cu_3_P/MoP poststability on Ni foam, and (f) full‐cell stability measured in a zero‐gap electrolyzer in 1 M alkaline seawater.

To experimentally verify the proposed mechanism, the long‐term durability of the catalyst was examined through chronopotentiometry measurements at a current density of 100 mA cm^−2^ in 1 M alkaline seawater (Figure 4a). A‐Cu_3_P/MoP exhibited outstanding stability, continuously sustaining HER activity for 500 h without any noticeable degradation. By contrast, the Cu_3_P/MoP electrode showed a gradual increase in potential and failed within 150 h under the same conditions. While Pt/C lost its activity within only a few hours under identical conditions, highlighting the superior durability of the ammonium‐modified catalyst in harsh seawater environments. Moreover, A‐Cu_3_P/MoP catalyst exhibited 100 h stability in natural seawater at 100 mA/cm^−2^ current density (Inset of Figure 4a).

The optical images of the electrodes after 100 h stability in natural seawater at 100 mA/cm^−2^ current density (Figure 4b) reveal a surface covered with dense, white precipitates over Pt/C, whereas A‐Cu_3_P/MoP (Figure 4c) retains a relatively clean and open porous morphology, indicating minimal scale buildup. The enhanced stability of the A‐Cu_3_P/MoP can be directly correlated to its anti‐precipitation capability, a major challenge in practical seawater electrolysis. Inductively coupled plasma mass spectrometry (ICP‐MS) analysis of the precipitate on the electrode surface (100 h stability in natural seawater at 100 mA/cm^−2^) revealed significant deposition of seawater‐derived cations on Pt/C, with 120 ng cm^−2^ of Mg^2+^ and 80 ng cm^−2^ of Ca^2+^ detected. In comparison, the A‐Cu_3_P/MoP displayed <4 ng cm^−2^ Mg^2+^ and no detectable Ca^2+^, a reduction of approximately 30‐fold in Mg^2+^ deposition, confirming its ability to suppress precipitation during prolonged electrolysis. In Figure 4d, Mg 1s ex situ XPS analysis of A‐Cu_3_P/MoP didn’t show magnesium precipitation, whereas on Pt/C, depositions were observed. Consistently, ex situ X‐ray diffraction patterns (Figure 4e) of the postelectrolysis of Pt/C electrode showed distinct crystalline peaks corresponding to magnesium hydroxide depositions [9, 22], whereas no such precipitate‐related peaks were observed for the A‐Cu_3_P/MoP electrode. Moreover, full‐cell seawater splitting was evaluated in a zero‐gap electrolyzer using a two‐electrode configuration, with A‐Cu_3_P/MoP as the cathode and FeCoF/FBT is an in‐house, solvothermally synthesized Fe—Co—MOF‐derived OER catalyst (Note S1), as the anode (Figure 4f). The corresponding polarization curve (Figure S15) confirms the ability to sustain a stable current density of ~420 mA cm^−2^ at 1.23 V for over 50 h without noticeable decay, demonstrating the durability of the ammonium‐modified interface under practical 1 M alkaline seawater operating conditions. In the zero‐gap configuration, the A‐Cu_3_P/MoP cathode is pressed against the membrane so that seawater passes through a thin NH_4_^+^ modified interfacial layer, minimizing OH^−^ build‐up and electrostatically repelling Mg^2+^/Ca^2+^, thereby suppressing cathodic precipitation even at high current density. Notably, the absence of performance loss under high‐current operation is consistent with the suppressed cathodic precipitation observed in half‐cell studies, further highlighting the potential of interfacial ammonium regulation for long‐term seawater electrolysis. These results clearly demonstrate that ammonium ion modification not only enhances the intrinsic catalytic activity of Cu_3_P/MoP but also imparts a robust anti‐precipitation capability, enabling sustained operation in seawater. Compared to the rapid deactivation of Pt/C, the ability of the A‐Cu_3_P/MoP to effectively resist precipitation by Ca^2+^ and Mg^2+^ species underscores its promise as a durable and practical HER catalyst for large‐scale seawater splitting applications.

**FIGURE 5:**
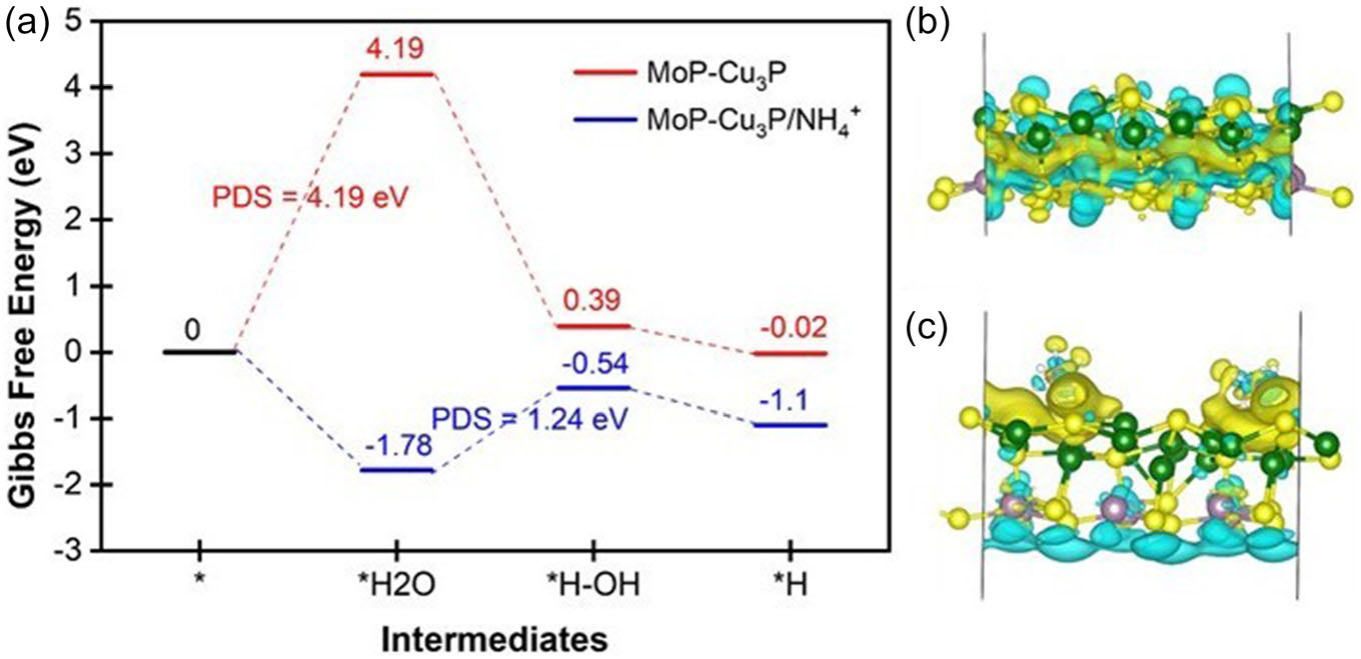
(a) Gibbs free energy plot for HER pathways, CDD plots of (b) Cu_3_P/MoP and (c) A‐Cu_3_P/MoP heterosurfaces. (Color code: yellow‐P, blue‐N, green‐Cu, metallic light violet‐Mo, and white‐H).

Gibbs free energy profiles provide insight into the thermodynamic feasibility and kinetic constraints of elementary steps in electrocatalytic hydrogen production. An optimal catalyst usually exhibits near‐zero *Δ* *G* for critical hydrogen intermediates. In this investigation, the alkaline HER route on Cu_3_P/MoP and A‐Cu_3_P/MoP was examined by creating Gibbs free energy diagrams for the sequence of adsorbed intermediates, i.e., *H_2_O, *H—OH, and *H (Figure 5a). The pristine Cu_3_P/MoP surface shows a significant uphill for the adsorption of H_2_O, with a potential‐determining step (PDS) of 4.19 eV.

Also, the plot indicates a severe thermodynamic barrier due to interfacial water dissociation and slow HER kinetics. In contrast, introducing NH_4_^+^ dramatically decreases the overall free energy of all intermediates and reduces the PDS to 1.24 eV at the *H—OH step, demonstrating the promoting function of NH_4_^+^ in water activation and proton transfer. The terminal *H state on A‐Cu_3_P/MoP becomes significantly more exergonic (−1.10 eV) compared to the nearly thermoneutral value (−0.02 eV) on pristine Cu_3_P/MoP. This highlights the enhanced thermodynamic driving force for hydrogen adsorption and subsequent evolution on the NH_4_^+^‐modified catalyst.

A thorough understanding of electronic structure and interfacial charge redistribution is required to explain the HER activity trends of TMPs on the heterosurface (Figure 2e,f). DFT PDOS analysis shows that NH_4_^+^ introduction causes N_p_ and H_s_ states to hybridize with Cu_d, Mo_d, and P_p near the Fermi level, broadening the electronic distribution and enhancing conductivity. Charge‐density‐difference isosurfaces further reveal NH_4_^+^ → Mo–P charge donation, corroborating XPS‐derived interfacial electronic enrichment that promotes faster HER kinetics.

The charge density difference isosurfaces in the A‐Cu_3_P/MoP system show significant charge donation from NH_4_^+^ to the catalyst surface, while electron accumulation (blue region) and depletion (yellow region) are mostly limited to the metal–P framework, as shown in Figure 5b,c. The NH_4_^+^‐induced charge redistribution changes the electronic structure and polarity of active centers, facilitating water dissociation and stabilizing critical hydrogen intermediates during the HER process. A detailed mechanistic illustration is provided in Figure S16.

## 4 Conclusion

In this work, we demonstrated a surface engineering strategy to suppress cathodic precipitation and enhance hydrogen evolution in alkaline seawater by constructing an ammonia‐modified Cu_3_P/MoP heterostructure. Structural analyses confirm that ammonium modification preserves the Cu_3_P/MoP nanosheet framework while introducing surface‐confined NH_4_^+^ species that modulate the interfacial electronic structure, particularly at Mo centers. DFT calculations reveal that this interfacial regulation lowers the water dissociation barrier and facilitates hydrogen intermediate stabilization, thereby accelerating HER kinetics. A‐Cu_3_P/MoP delivers outstanding HER performance, achieving 0.50 A cm^−2^ at 574 mV in natural seawater and 1.0 A cm^−2^ at 416 mV in alkaline seawater, outperforming commercial Pt/C. The catalyst exhibits a low Tafel slope (33.5 mV dec^−1^), reduced charge‐transfer resistance, and an enlarged ECSA, evidencing rapid charge transfer and a high density of accessible active sites. Crucially, A‐Cu_3_P/MoP maintains stable operation for over 500 h at 100 mA cm^−2^ in alkaline seawater and 100 h in natural seawater, whereas Pt/C rapidly deactivates due to Mg(OH)_2_ and Ca(OH)_2_ deposition. Moreover, full‐cell seawater splitting was evaluated in a zero‐gap electrolyzer with working area of 25 cm^2^, with A‐Cu_3_P/MoP against the lab designed anode. The system sustained a stable current density of ~420 mA cm^−2^ at 1.23 V for over 50 h without noticeable decay, demonstrating the durability of the ammonium‐modified interface under practical operating conditions. Overall, this study establishes interfacial ammonium regulation as a powerful strategy to simultaneously tune electronic structure, enhance HER kinetics, and mitigate cathodic precipitation in seawater electrolytes.

## Conflicts of Interest

The authors declare no conflicts of interest.

## Supporting information

Supplementary Material

## Data Availability

The data that support the findings of this study are available on request from the corresponding author. The data are not publicly available due to privacy or ethical restrictions.
