# Supplementary material for: A Surface Engineering Strategy to Suppress Cathodic Precipitation in Seawater Electrolysis
Source: Small Sci. 2026 Aug 12;6(8):e70368. doi: 10.1002/smsc.70368 (PMC13489221; doi:10.1002/smsc.70368)
Supplement: Supplementary file 1 [file SMSC-6-e70368-s001.pdf]

Supplementary Information

A Surface Engineering Strategy to Suppress Cathodic Precipitation in Seawater Electrolysis

Vasundhara Nettem[a], Muhammad Waqas Khan[a], Suraj Loomba[a], Sharafadeen Gbadamasi[a], Tushar Verma[a,b], Babar Shabbir[a], Ravichander Babarao[a]*, Nasir Mahmood[a]*

[a] School of Science, RMIT University, Melbourne, Victoria 3001, Australia

[b] Academy of Scientific and Innovative Research (AcSIR), Ghaziabad, 201002, India

2.1 Materials

Copper(II) nitrate trihydrate ($Cu(NO_3)_2 \cdot 3H_2O$); Ammonium molybdate tetrahydrate ($(NH_4)_6Mo_7O_{24} \cdot 4H_2O$); Sodium hypophosphite (NaH_2PO_2); Ammonium hydroxide (NH_4OH); Sodium hydroxide (NaCl); Polytetrafluoroethylene (PTFE) (Sigma); Isopropyl alcohol (Sigma); Absolute ethanol (Sigma).

Materials Characterizations

X-ray diffraction (XRD) pattern was recorded using Bruker D4 Endeavor to obtain the crystal structures of the as-synthesised materials. Transmission electron microscope (TEM), high-resolution TEM (HR-TEM), selected area electron diffraction (SAED), and energy dispersive X-ray spectroscopy (EDS) were obtained using a JEOL 2100F to analyse the morphology, crystal structure, and lattice parameters. A LabRAM HR Evolution Raman spectrometer (Horiba Scientific) was used to obtain the Raman spectra under a 532 nm laser with a power of 50 mW. A Thermo Scientific K-alpha system was used to conduct high-resolution X-ray photoelectron spectroscopy (XPS). The system consists of an Al Kα monochromated X-ray source for scanning the samples at a dwell time of 50 ms^{-1} and pass time of 50 eV. The Agilent inductively coupled plasma mass spectrometer (ICP-MS) with laser ablation 2 capability was used to acquire the ICP-MS results.

Computational Details

The spin-polarized Kohn Sham Generalised DFT method has been used for all geometry optimizations and energy calculations.[9–11] The generalized DFT technique, with Grimme semi-empirical (DFT-D3) corrections, was used to include Van der Waals forces in all systems.[12] DFT+ U adjustments with U = 5 for the Cu transition metal in the ground state geometry.[13,14] The exchange-correlation energy has been

defined using the generalized gradient approximation in the Perdew Burke Ernzerhof (PBE) functional, which is implemented in the Vienna Ab initio Simulation Package (VASP) program. The Projected Augmented Wave (PAW) method has been utilized to describe electron ion interactions.[15] Following benchmarking, a 520 eV kinetic energy cut-off is taken into consideration for a plane wave basis. A vacuum of 30 Å along the z-axis is used for all geometry and frequency computations, and a 3 x 3 x 1 Monkhorst–Pack k-point grid is taken into consideration to prevent any undesired interlayer interactions. Further, for electronic properties calculations such as Density of States (DOS) and Charge Density Difference (CDD) evaluation, a 11 x 11 x 1 Monkhorst-Pack k-point system has been used. Convergence thresholds were set at 10^{-7} eV for electronic energy changes and 0.005 eV/Å for structural optimizations.

The H_2O adsorption energies (E_a) on Cu_3P-MoP and Cu_3P-MoP/NH_4^+ heterosurfaces are evaluated by the equation-

$$E_a = E_{\text{heterosurface}_{H2O}} - E_{heterosurface} - E_{H2O} \qquad (1)$$

Where, $E_{\text{heterosurface}_{H2O}}$ is total electronic energy of H_2O adsorbed on Cu_3P-MoP and Cu_3P-MoP/NH_4^+ heterosurfaces , $E_{heterosurface}$ is energy of Cu_3P-MoP and Cu_3P-MoP/$NH_4^{+\,+}$ heterosurfaces , E_{H2O} is energy of free H_2O.

The Gibbs free energy change (ΔG) of each elementary catalytic intermediate step involved in NRR and HER was evaluated using the computational hydrogen electrode (CHE) model given by Nørskov *et al.*

$$\Delta G = EE + ZPE - T\Delta S \qquad (2)$$

Where EE is electronic energy obtained from optimization calculations, ZPE is change in zero-point energy, T is temperature (298.15K) and ΔS is change in entropy, both of which are obtained from frequency calculations. EE, ZPE and ΔS are calculated using vaspkit.1.3.0 software.

Reaction mechanisms involved-

HER

$$* + H_2O + e^- \rightarrow H^* + OH^- \quad \text{……(3)}$$

Table ST1. Electronic energies, ZPE, TS, and change in Gibbs Free Energy (ΔG) (in eV) of HER intermediates for MoP-Cu_3P heterosurface.

PDS = 4.19 eV

Intermediates	Electronic Energy	ZPE	TS	G	ΔG
*H_2O	-220.53	0.22	0.16	-220.47	4.19
*H-OH	-224.58	0.43	0.11	-224.27	0.39
*H	-213.93	0.14	0.01	-213.8	-0.002

Table ST2. Electronic energies, ZPE, TS, and change in Gibbs Free Energy (ΔG) (in eV) of HER intermediates for Cu_3P-MoP/NH_4^+ heterosurface.

PDS = 1.24 eV

Intermediates	Electronic Energy	ZPE	TS	G	ΔG
*H_2O	-301.08	0.49	0.11	-300.71	-1.78
*H-OH	-299.83	0.47	0.10	-299.47	-0.54
*H	-289.26	0.14	0.03	-289.15	-1.10

Figure S1. Ground State Geometries of HER intermediates for MoP-Cu_3P heterosurface. (Color Code – Yellow- P, Blue-N, Green-Cu, Metallic light violet-Mo, Red-O and Cyan-H)

Supplementary figures:

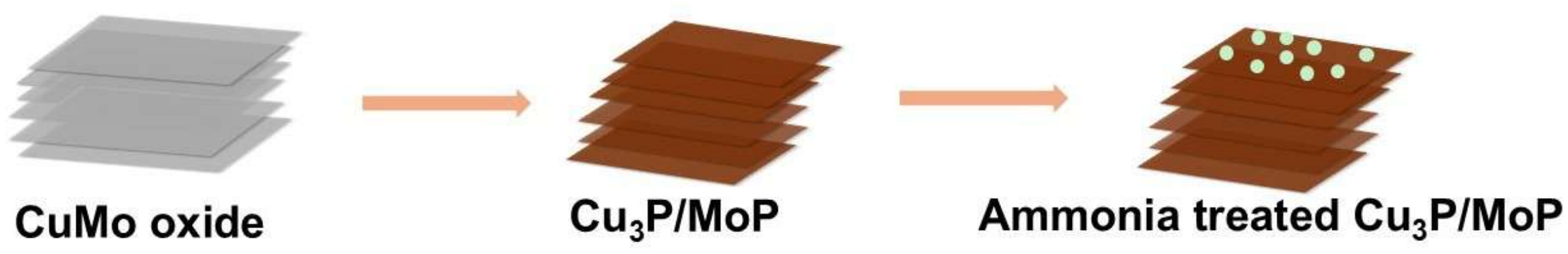

Supplementary Figure 1: schematic illustration of the synthesis process.

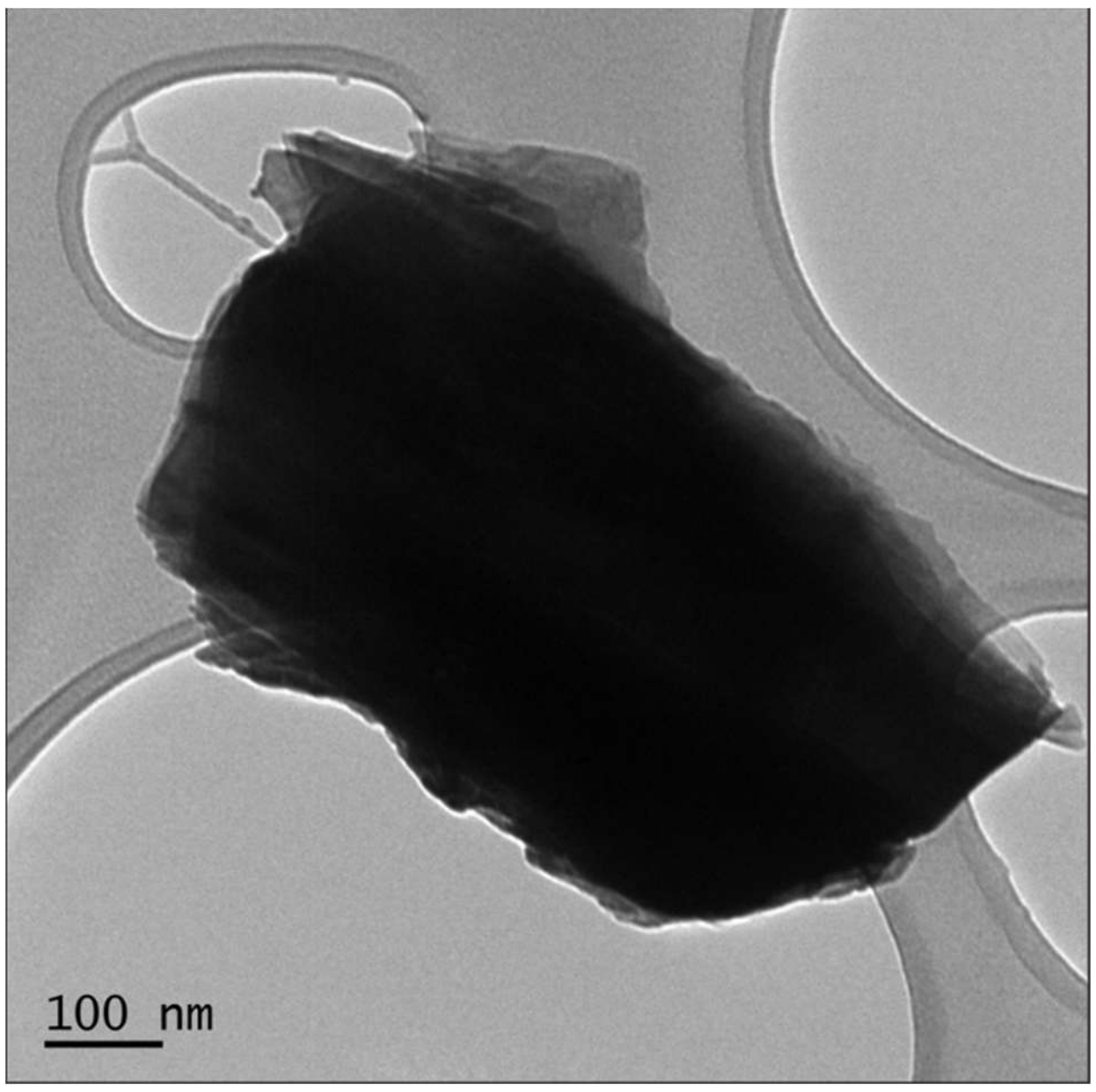

Supplementary Figure 2: TEM image of CuO

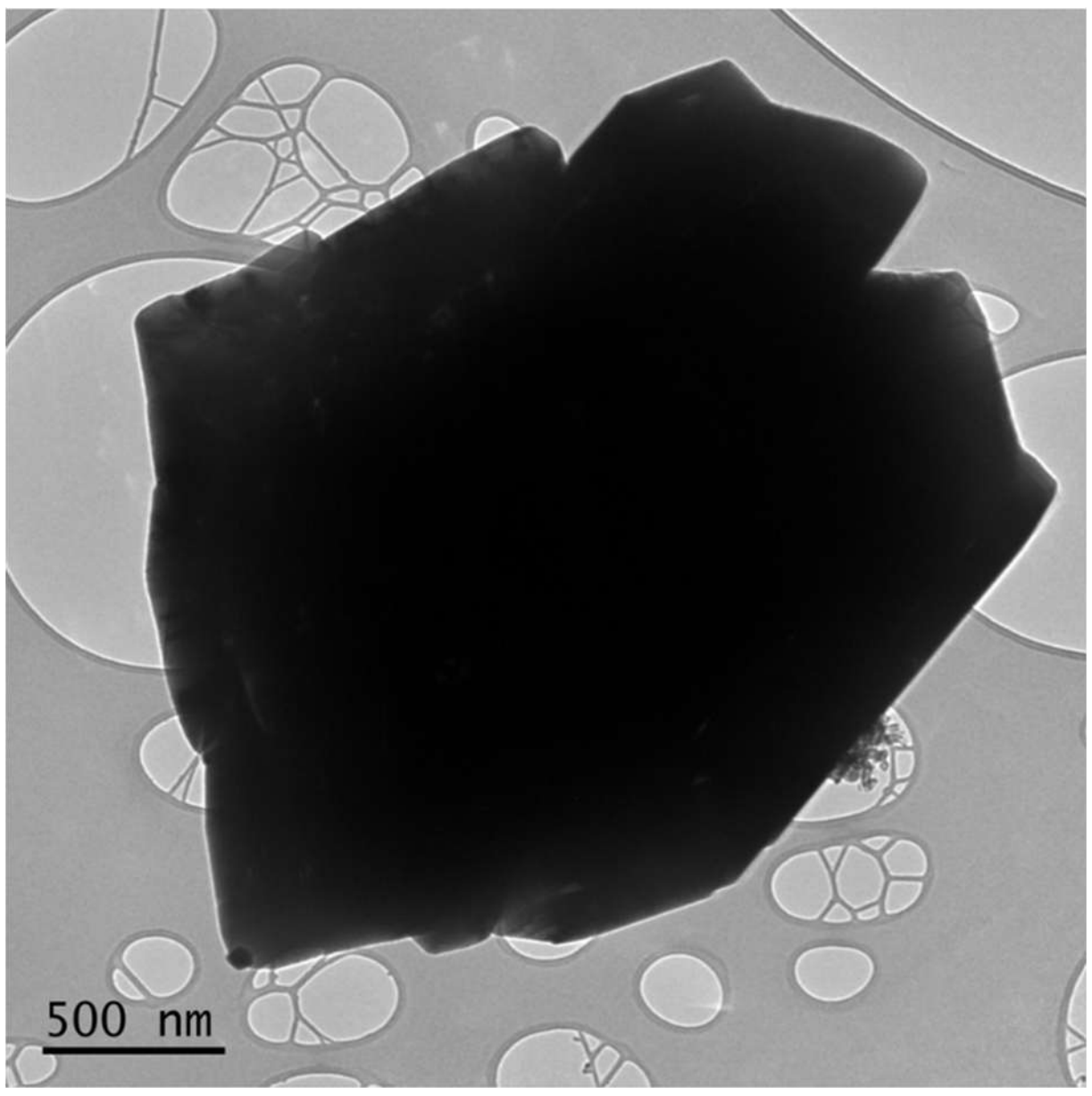

Supplementary Figure 3: TEM image of CuP

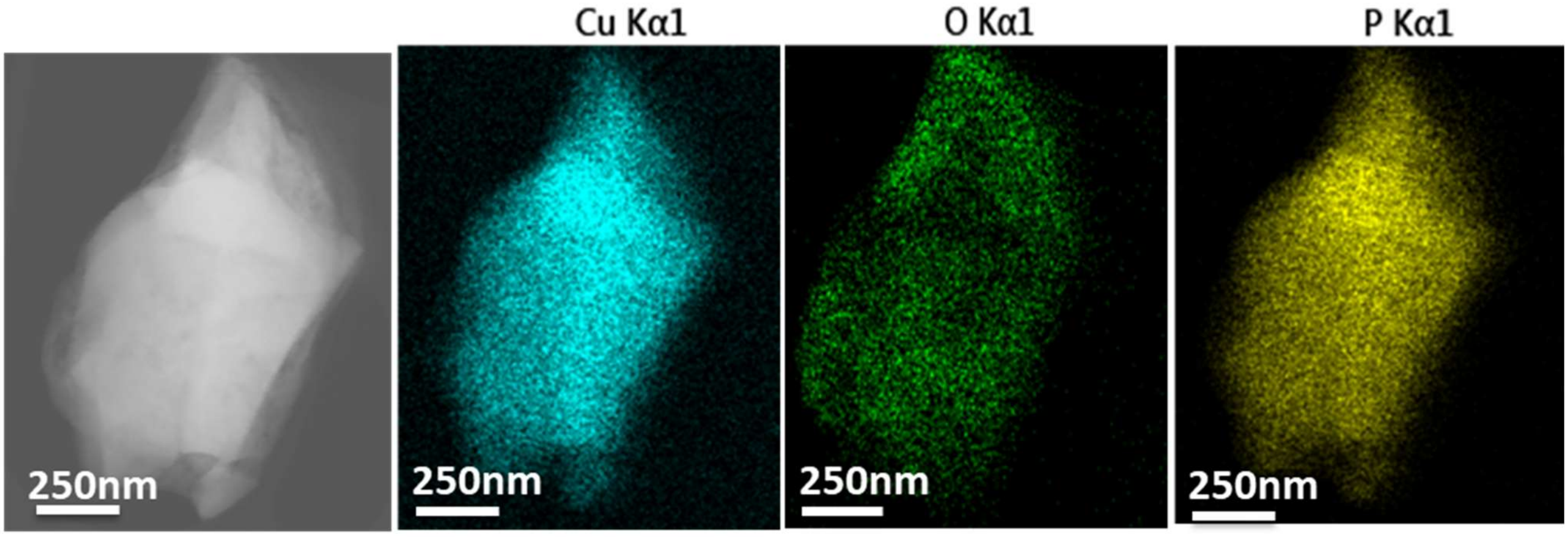

Supplementary Figure 4: Energy-dispersive X-ray spectroscopy (EDS) of CuP showing uniform distribution of elements across the sheets.

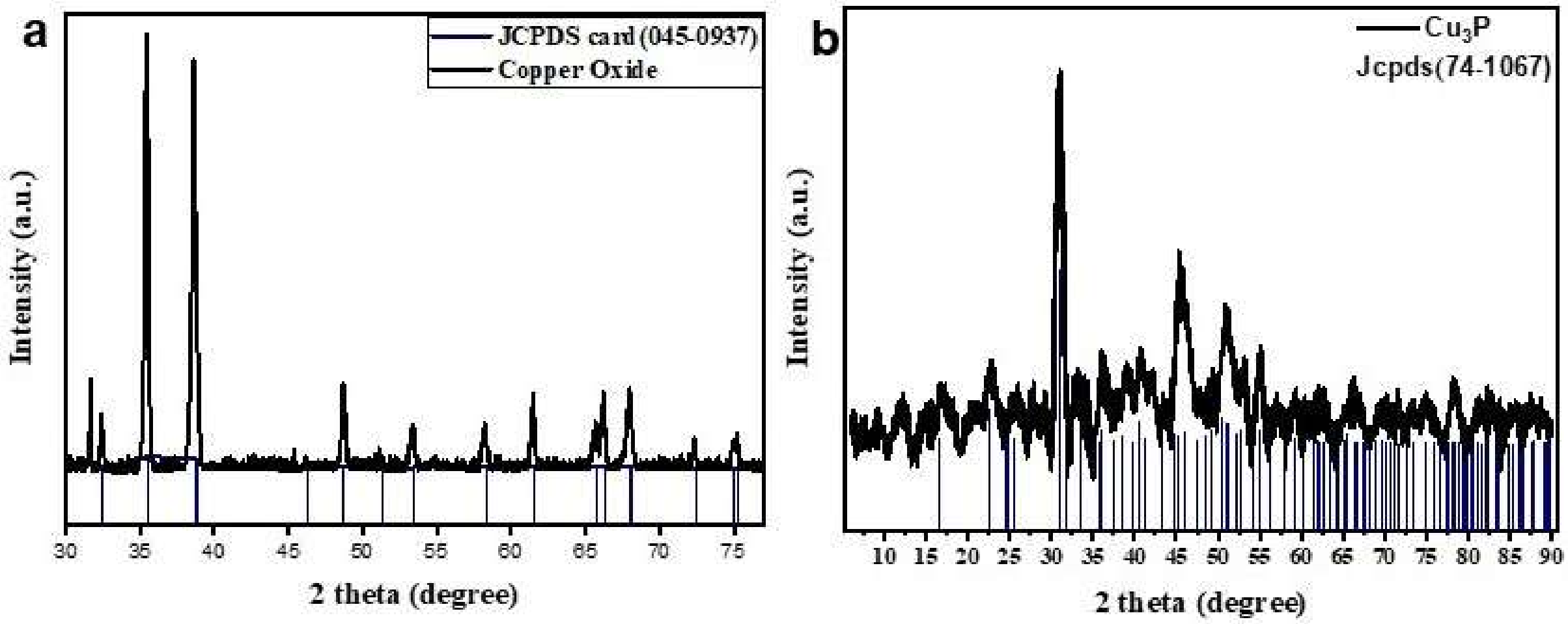

Supplementary figure 5: XRD pattern of a) CuO, b) Cu_3P

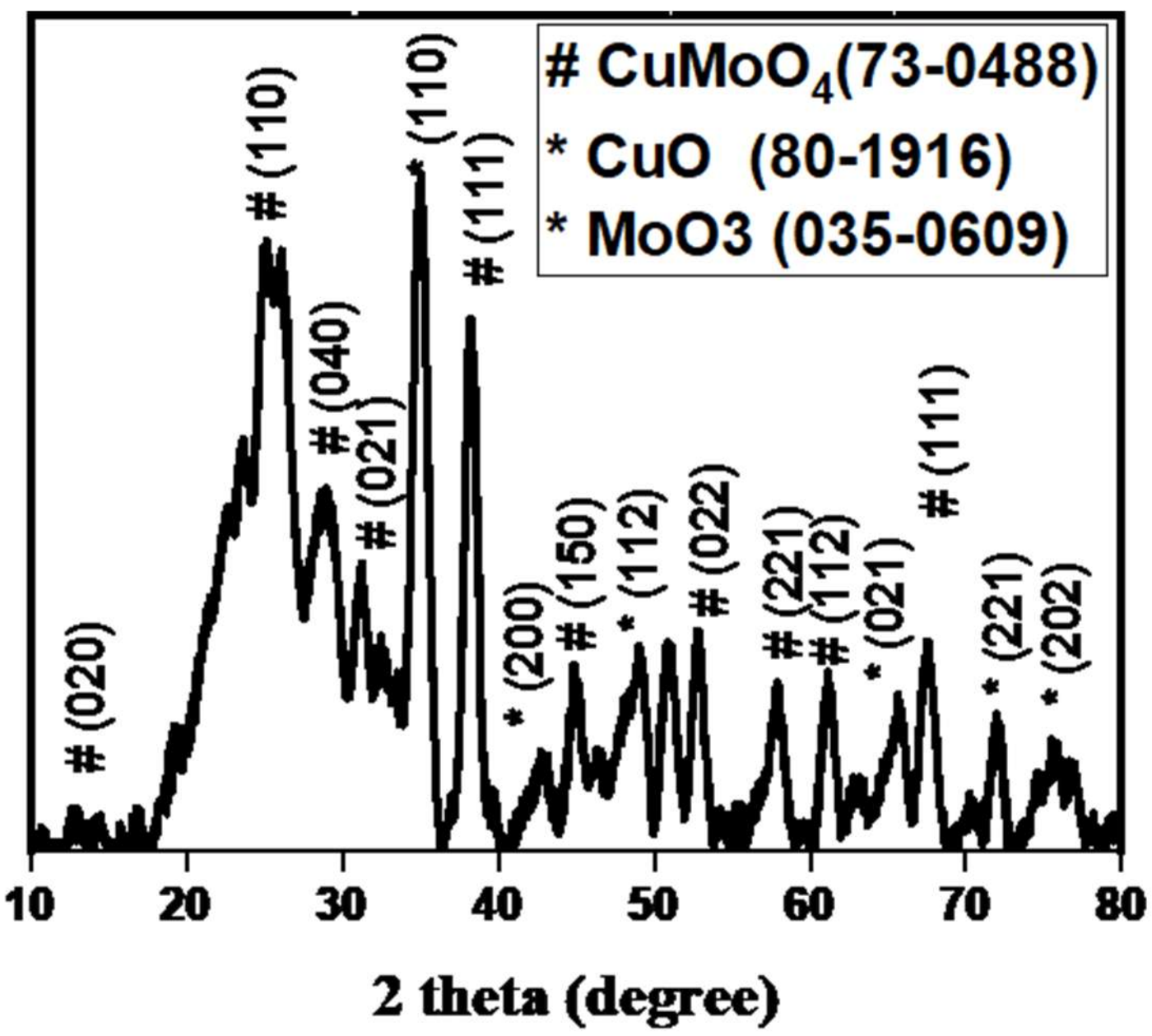

Supplementary figure 6: XRD pattern of $CuMoO_4$

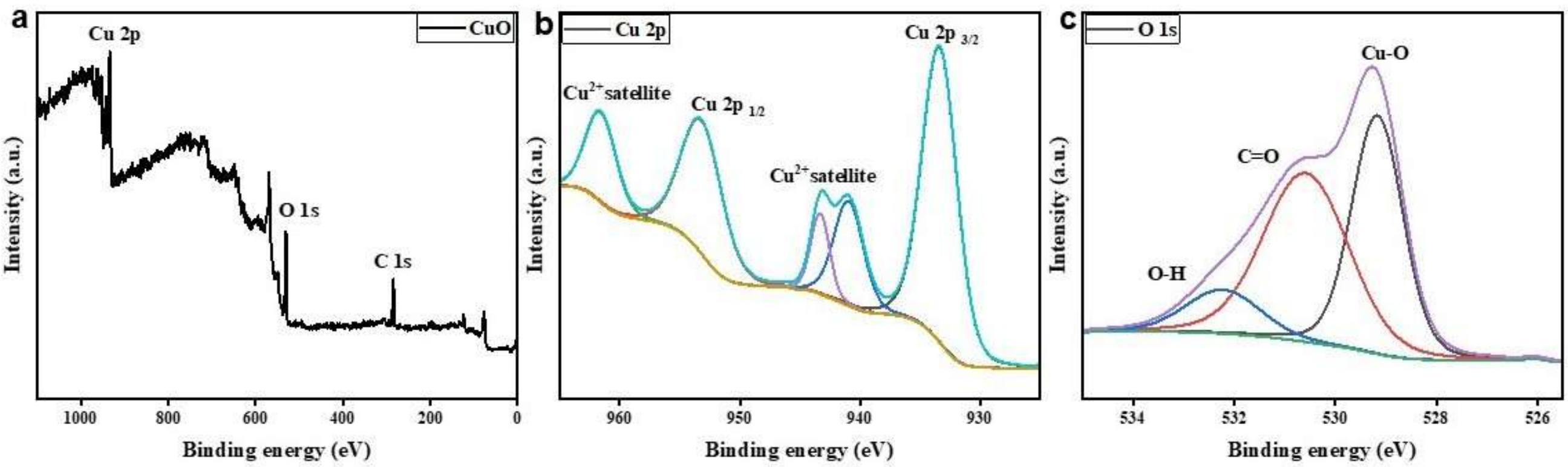

Supplementary figure 7: XPS of CuO a) Survey spectra b) Cu2p c) O1s

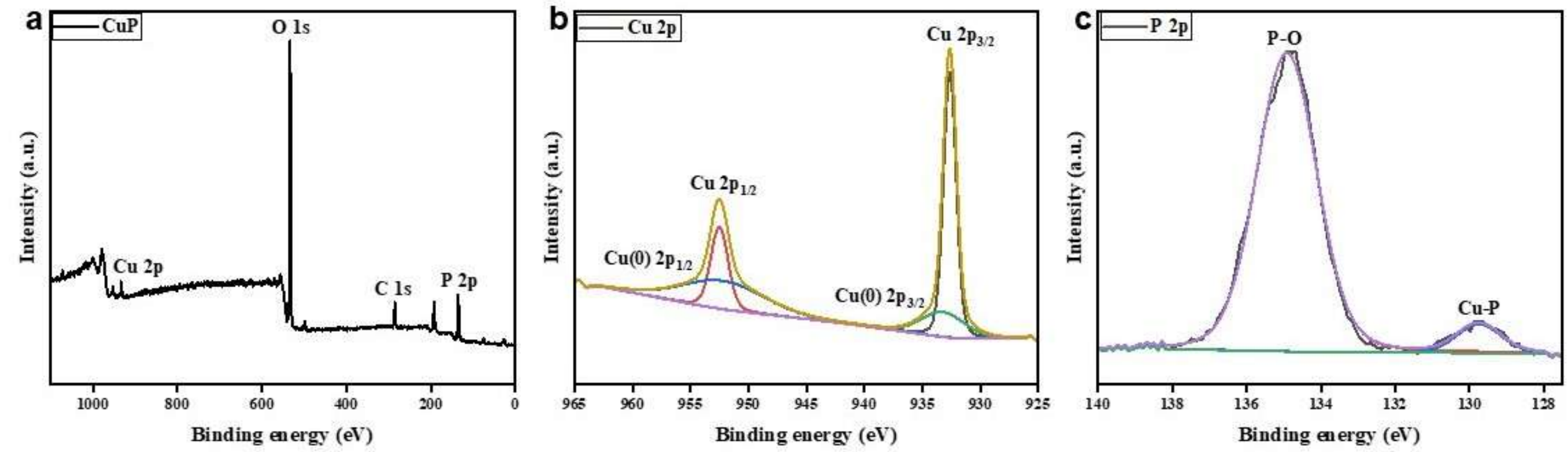

Supplementary figure 8: XPS of CuP a) Survey spectra b) Cu2p, c) P2p

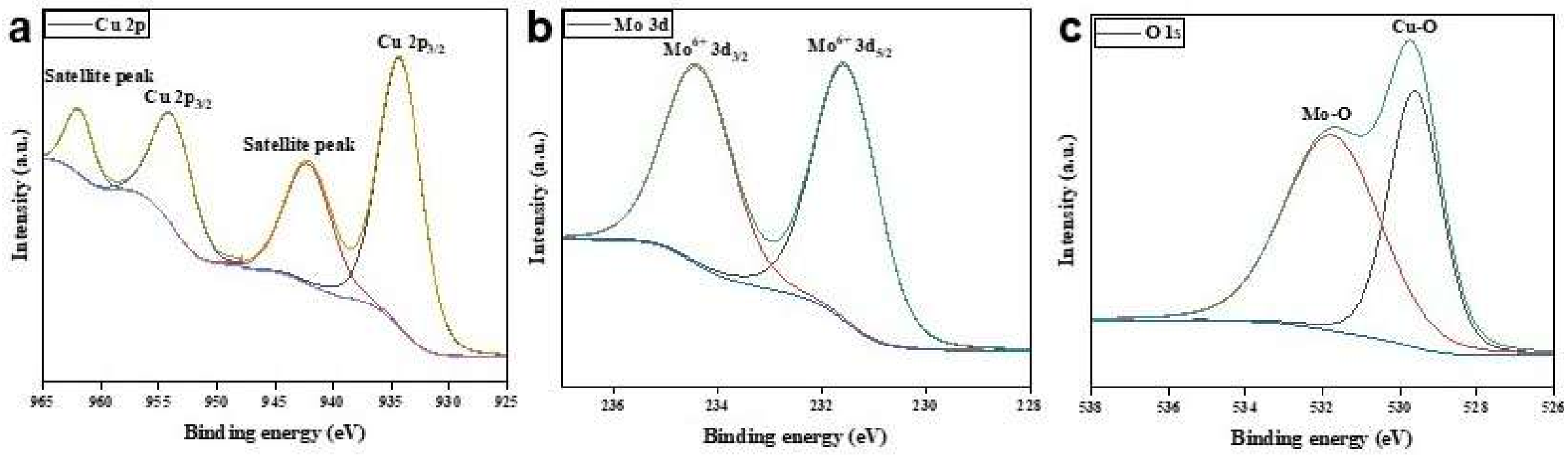

Supplementary Figure 9: XPS of $CuMoO_4$ a) Cu 2p, b) Mo 3d, c) O 1s

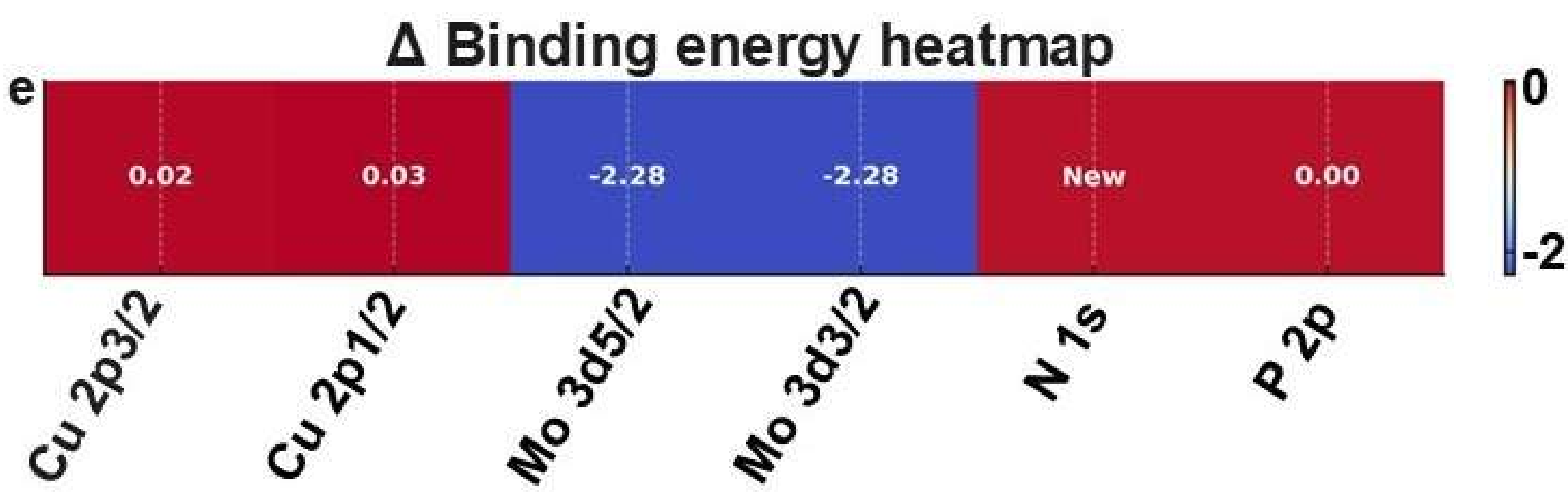

Supplementary Figure 10: Binding energy heat map

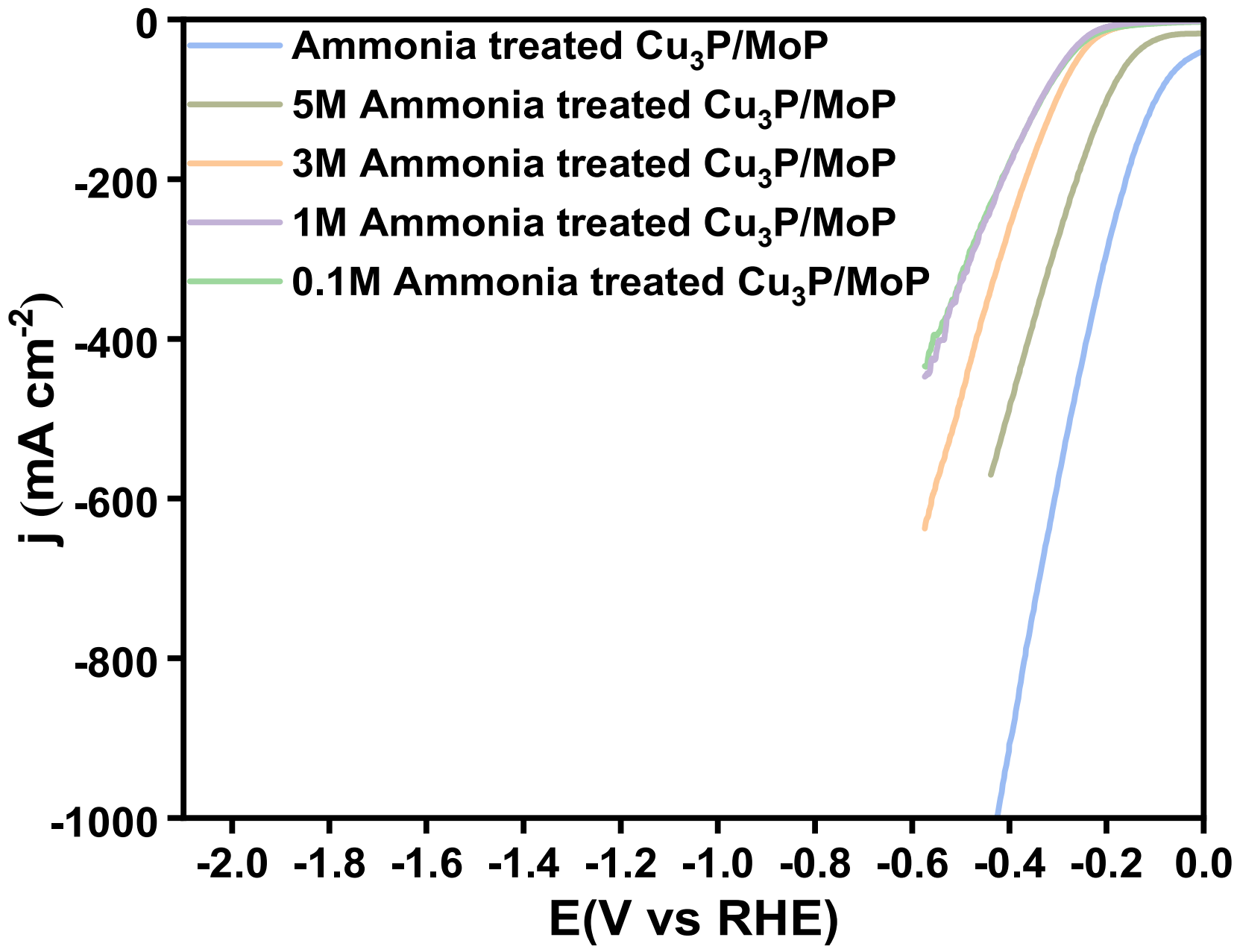

Supplementary Figure 11: LSV of different concentrations of ammonia-treated Cu_3P/MoP.

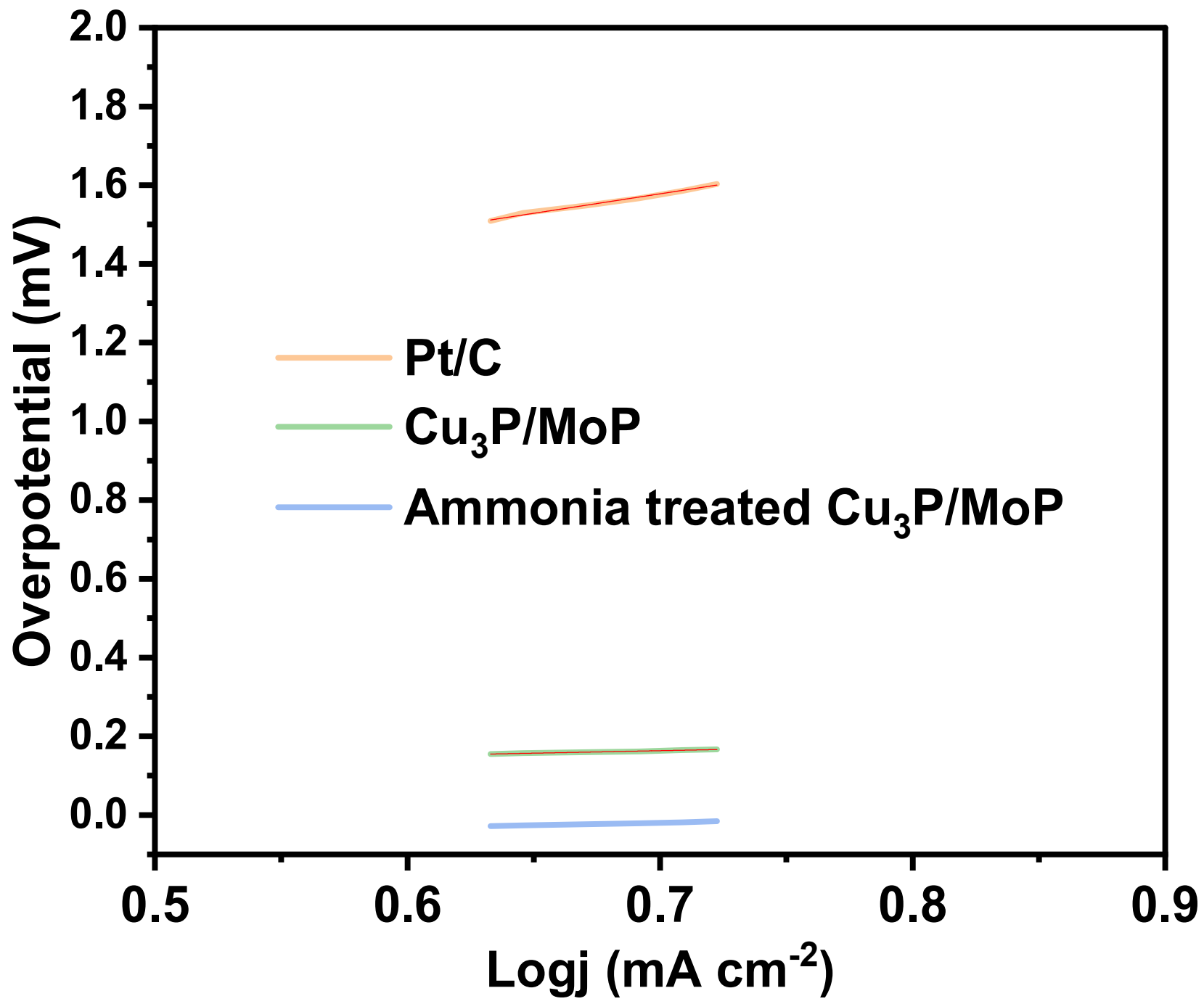

Supplementary Figure 12: Tafel slopes of the electrocatalysts from the polarisation curve A-Cu_3P/MoP, Cu_3P/MoP, Pt/C.

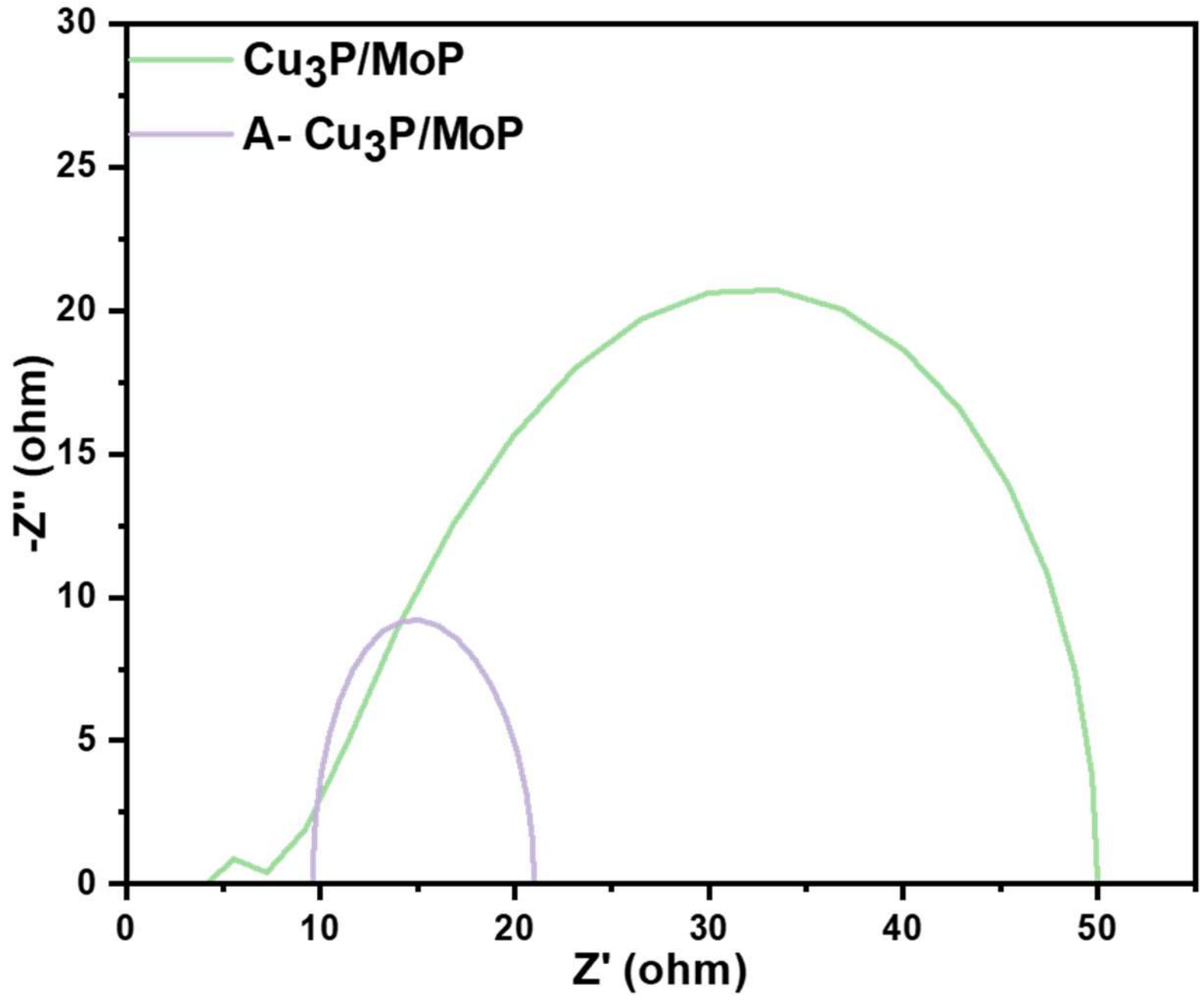

Supplementary Figure 13: EIS of of A-Cu_3P/MoP, Cu_3P/MoP

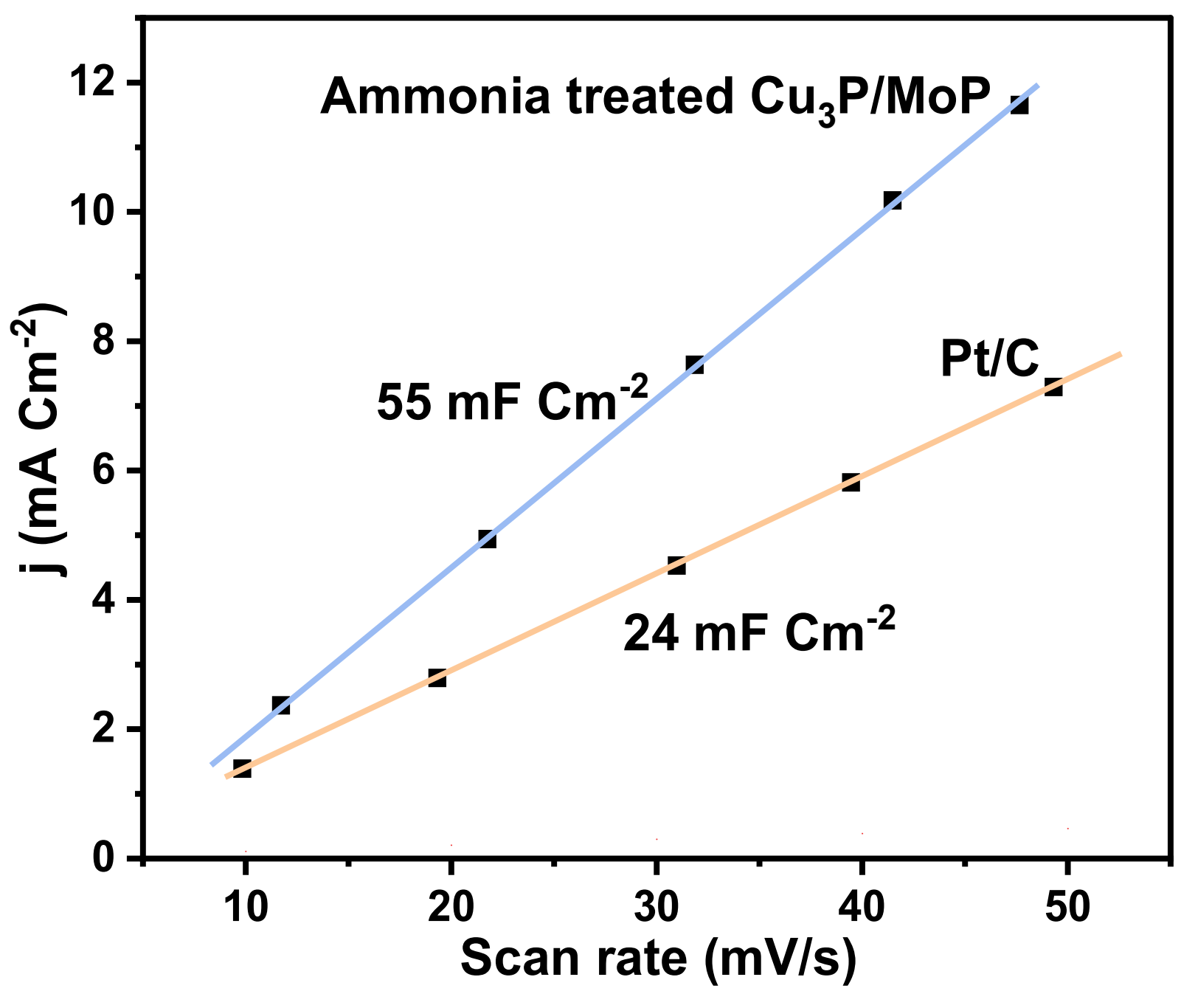

Supplementary Figure 14: Double layer capacitance (C_{dl}) curves of A-Cu_3P/MoP, Pt/C.

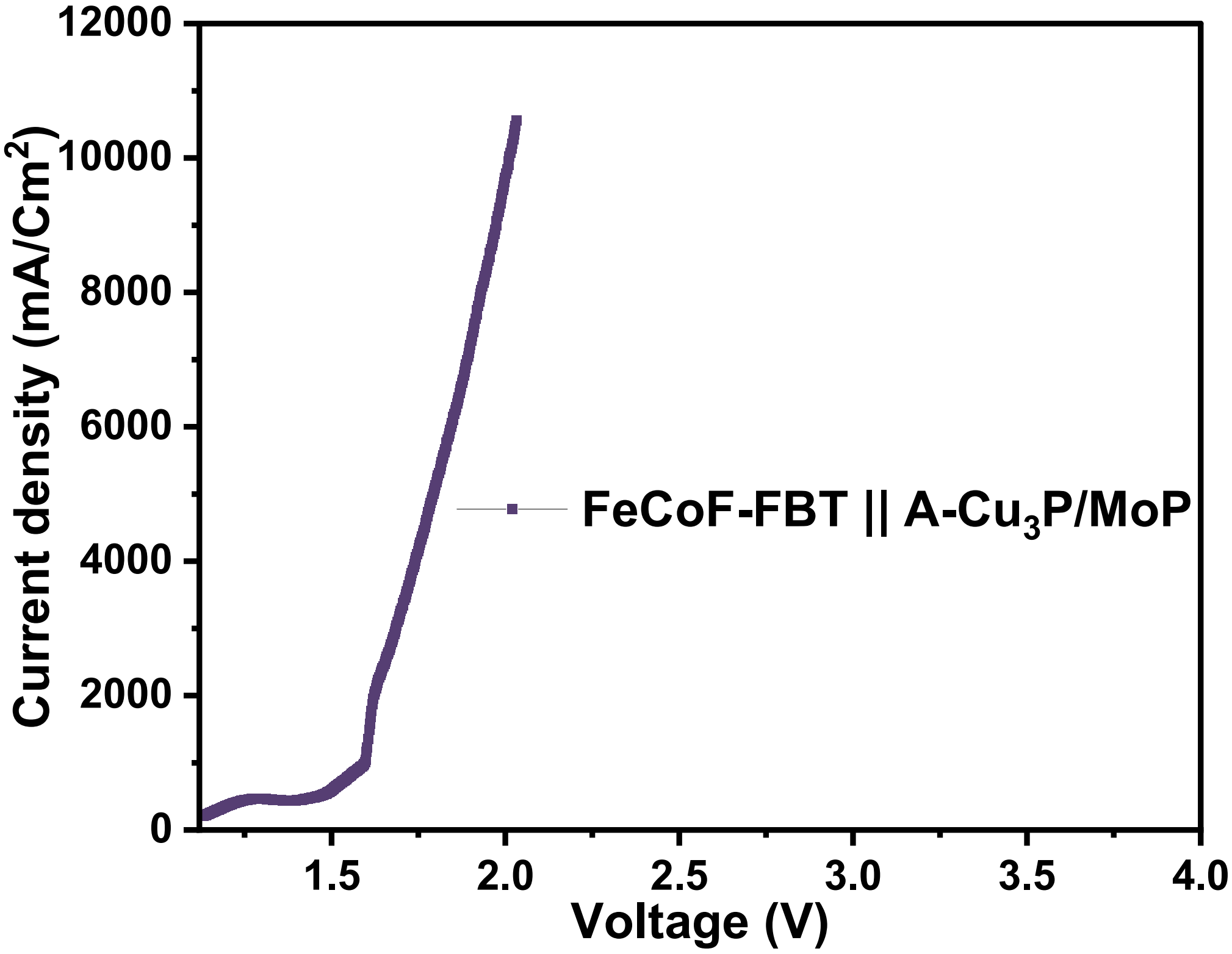

Supplementary Figure 15: Polarisation curve of FeCoF-FBT || A-Cu_3P/MoP in zerogap electrolyser.

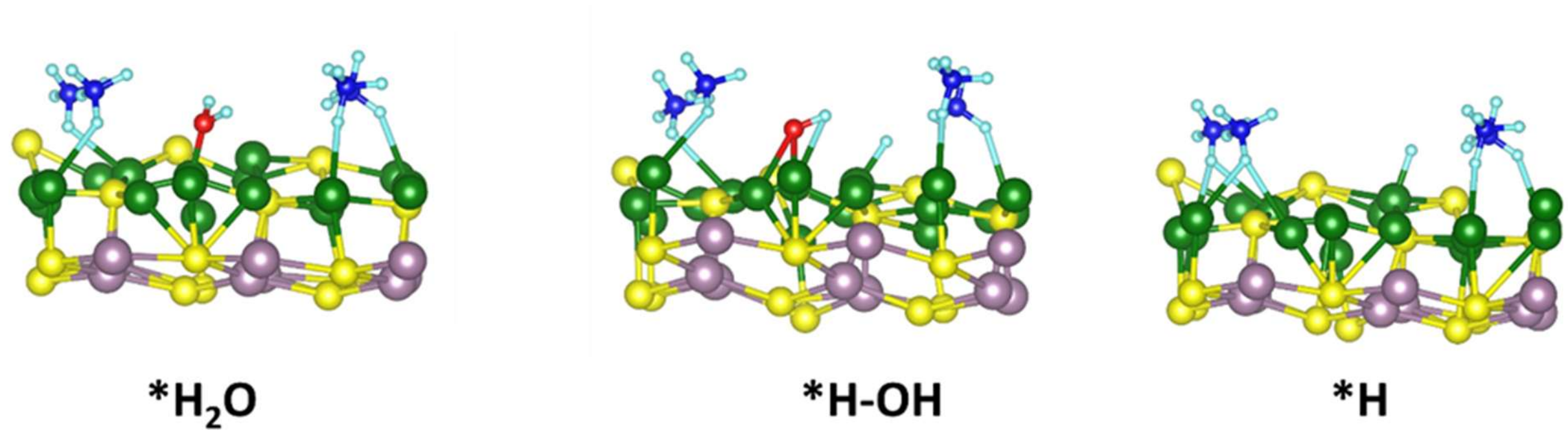

Supplementary Figure 16. Ground State Geometries of HER intermediates for A-Cu_3P/MoP heterosurface. (Color Code – Yellow- P, Blue-N, Green-Cu, Metallic light violet-Mo, Red-O, and Cyan-H).

Supplementary note 1:

An in-house Fe–Co–MOF–based catalyst (FeCoF/FBT) developed in our laboratory was used as the OER anode in this work. The material was synthesised following our established solvothermal protocol, with compositions denoted FeCoF, FeCoF/FBT-10, FeCoF/FBT-15 and FeCoF/FBT-20 according to the FBT (NaOH-treated $Ti_3C_2T_x$ with Fe and B precursors) content.

References

1 Z. Zhang, G. Yu, H. Li, J. Liu, X. Huang and W. Chen, *Physical Chemistry Chemical Physics*, 2018, 20, 10407–10417.

2 C. Yang, Y. Chen, Z. He, R. Li and X. Gou, *Front. Chem.*, DOI:10.3389/fchem.2025.1629904.

3 Z. Zhang, G. Yu, H. Li, J. Liu, X. Huang and W. Chen, *Physical Chemistry Chemical Physics*, 2018, 20, 10407–10417.

4 X. Tian, T. Wang, L. Fan, Y. Wang, H. Lu and Y. Mu, *Appl. Surf. Sci.*, 2018, 427, 357–362.

5 X. Huang, J. Xu, X. Xu and S. Wang, *ACS Catal.*, 2024, 14, 14606–14621.

6 M. Rafiq, S. U. Rahman, W. Xu, H. Xu, O. H. Abbasi, N. Kitchamsetti, G. Gahungu, W. Li and J. Zhang, *Electrochim. Acta*, 2026, 548, 147967.

7 M. Schalenbach, R. Tesch, P. M. Kowalski and R.-A. Eichel, *Physical Chemistry Chemical Physics*, 2024, 26, 14171–14185.

8 R. Yao, K. Sun, K. Zhang, Y. Wu, Y. Du, Q. Zhao, G. Liu, C. Chen, Y. Sun and J. Li, *Nat. Commun.*, 2024, 15, 2218.

9 J. A. Pople, P. M. W. Gill and B. G. Johnson, *Chem. Phys. Lett.*, 1992, 199, 557–560.

10 J. Moellmann and S. Grimme, *The Journal of Physical Chemistry C*, 2014, 118, 7615–7621.

11 L. Goerigk, in *Non-Covalent Interactions in Quantum Chemistry and Physics*, Elsevier, 2017, pp. 195–219.

12 J. Hafner, *J. Comput. Chem.*, 2008, 29, 2044–2078.

13 R. Tesch and P. M. Kowalski, *Phys. Rev. B*, 2022, 105, 195153.

14 E. Macke, I. Timrov, N. Marzari and L. C. Ciacchi, *J. Chem. Theory Comput.*, 2024, 20, 4824–4843.

15 K. F. Garrity, J. W. Bennett, K. M. Rabe and D. Vanderbilt, *Comput. Mater. Sci.*, 2014, 81, 446–452.
